# Association of serum 25-hydroxyvitamin D with sleep disorders in individuals with prediabetes and diabetes: a prospective cohort study

**DOI:** 10.3389/fendo.2025.1524368

**Published:** 2025-04-01

**Authors:** Guodong Liu, Huaxue Li, Yicheng Ma, Yingzhou Shi, Junming Han, Fei Li, Li Fang, Shengyu Tian, Yiping Cheng

**Affiliations:** ^1^ Department of Endocrinology, Shandong Provincial Hospital Affiliated to Shandong First Medical University, Jinan, Shandong, China; ^2^ Shandong Clinical Research Center of Diabetes and Metabolic Diseases, Jinan, Shandong, China; ^3^ Department of Endocrinology, Shandong Provincial Hospital, Shandong University, Jinan, Shandong, China

**Keywords:** serum 25-hydroxyvitamin D, sleep disorders, prediabetes, diabetes, UK Biobank, adult

## Abstract

**Background:**

Sleep disorders are common globally and are linked to various adverse health outcomes, including cardiovascular disease, type 2 diabetes, and mental health conditions. Emerging evidence suggests a potential role of serum 25-hydroxyvitamin D (25(OH)D) in regulating sleep. Individuals with prediabetes and diabetes are at an increased risk of both vitamin D deficiency and sleep disorders, yet the relationship between these factors remains insufficiently studied. Utilizing data from the UK Biobank, this study aims to investigate the association between serum 25(OH)D concentrations and the risk of sleep disorders in individuals with prediabetes and diabetes.

**Methods:**

We conducted a cross-sectional study of 81,533 participants (mean age 59.6 [SD 7.1] years, comprising 41,275 males [50.6%] and 40,278 females [49.4%]) from the UK Biobank, followed by a prospective study of 80,546 participants (mean age 59.6 [SD 7.1] years, comprising 40,513 males [50.3%] and 40,033 females [49.7%]) within the same cohort, focusing on individuals with prediabetes and diabetes. Baseline serum 25(OH)D concentrations were categorized into four groups: severe deficiency (<25.0 nmol/L), moderate deficiency (25.0–50.0 nmol/L), insufficiency (50.0–75.0 nmol/L), and sufficiency (≥75.0 nmol/L). Multivariable logistic regression and Cox proportional hazards models were used, adjusting for demographic, lifestyle, and health-related confounders.

**Results:**

Over an average follow-up of 12.8 years, we documented 2,704 cases of sleep disorders and found that higher serum 25(OH)D concentrations were significantly associated with reduced sleep disorder risk. In fully adjusted models, sufficient serum 25(OH)D concentrations reduced sleep disorder risk by 48% in prediabetes (HR = 0.52; 95% CI: 0.41–0.65) and 52% in diabetes (HR = 0.48; 95% CI: 0.34–0.67). Subgroup analysis found that adequate vitamin D concentrations were associated with improved sleep health especially in people ≤60 years of age, women, BMI≥30kg/m², and those who had never smoked.

**Conclusion:**

This study provides strong evidence that higher serum 25(OH)D concentrations are associated with a reduced risk of sleep disorders in individuals with prediabetes and diabetes. These findings suggest that maintaining adequate vitamin D concentrations may offer a potential strategy to improve sleep health in this population.

## 1 Introduction

Sleep disorders, encompassing conditions such as insomnia, obstructive sleep apnea, periodic limb movement disorder, and narcolepsy, represent a widespread chronic health issue globally (1, 2). It is estimated that approximately 10% to 30% of adults worldwide experience sleep disorders of varying severity, with the prevalence of insomnia symptoms generally increasing with age (3). Chronic sleep disturbances not only reduce quality of life but also elevate the risk of cardiovascular disease, type 2 diabetes, and serious mental illnesses such as depression and anxiety. In addition to lifestyle, sleep environment, and psychosocial factors, serum 25-hydroxyvitamin D (25(OH)D) concentrations are believed to be closely related to the occurrence and development of sleep disorders.

As a fat-soluble vitamin, vitamin D plays a crucial role in regulating calcium-phosphorus metabolism, maintaining bone health, and balancing mineral levels (4). 25(OH)D is the primary circulating form of vitamin D in the body, and its level can reflect the nutritional status of vitamin D. Epidemiological evidence suggests that vitamin D deficiency is associated not only with musculoskeletal diseases (5), but also with an increased risk of various chronic conditions, including cardiovascular disease, autoimmune diseases, and cancer (6–9). Increasing evidence links vitamin D deficiency to a higher risk of psychiatric disorders such as sleep disturbances, depression, and anxiety (10, 11). A Mendelian randomization study based on NHANES data found that low 25(OH)D concentrations in the general adult population were significantly associated with an increased risk of sleep disorders, even after controlling for confounders (12). A meta-analysis also revealed a general association between vitamin D deficiency and sleep disorders in Asian populations (13).

Current studies provide preliminary evidence of the association between vitamin D and sleep disorders, primarily focusing on the general population. Global epidemiological data indicate that, despite recent declines in the incidence and mortality of cardiovascular diseases among individuals with prediabetes and diabetes, the prevalence of mental illnesses, including sleep disorders and depression, continues to rise yearly (14). However, research on the relationship between vitamin D deficiency and sleep disorders in individuals with prediabetes and diabetes remains limited, with insufficient systematic evidence. Additionally, compared to the general population, individuals with prediabetes and diabetes are often more prone to vitamin D deficiency, with lower serum 25(OH)D concentrations (15–18). This may result from factors such as insulin resistance, impaired kidney function, and drug interactions.

This study aims to utilize data from the UK Biobank, a large prospective cohort study, to explore the association between serum 25(OH)D concentrations and the risk of sleep disorders in individuals with prediabetes and diabetes. Our research incorporates both cross-sectional and prospective cohort designs. Additionally, we examine whether there is heterogeneity in the associations across different subgroups, such as age, sex, and smoking status. Through this study, we hope to provide new insights into the mechanisms by which vitamin D influences diabetes-related sleep disorders, and to offer a scientific basis for developing targeted clinical interventions to improve sleep quality in patients.

## 2 Materials and methods

### 2.1 Data availability

The UK Biobank is a large prospective cohort study conducted from 2006 to 2010, which recruited over 500,000 participants aged 40 to 69 years from 22 assessment centers across the United Kingdom (19). Upon recruitment, all participants provided written informed consent. Those who consented completed a touchscreen questionnaire and participated in face-to-face interviews, where sociodemographic, lifestyle, and health information was collected. Additionally, a series of physical examinations were conducted, and participants provided blood, urine, and saliva samples.

The UK Biobank received ethical approval from the North West Multi-centre Research Ethics Committee (MREC) as a “Research Tissue Bank” (RTB). The general ethical approval for UK Biobank research was granted by the National Research Ethics Service Centre (http://www.ukbiobank.ac.uk/ethics/). All participants provided written informed consent, and the study protocol is accessible online (http://www.ukbiobank.ac.uk/).

### 2.2 Study design and participants

**Figure 1:**
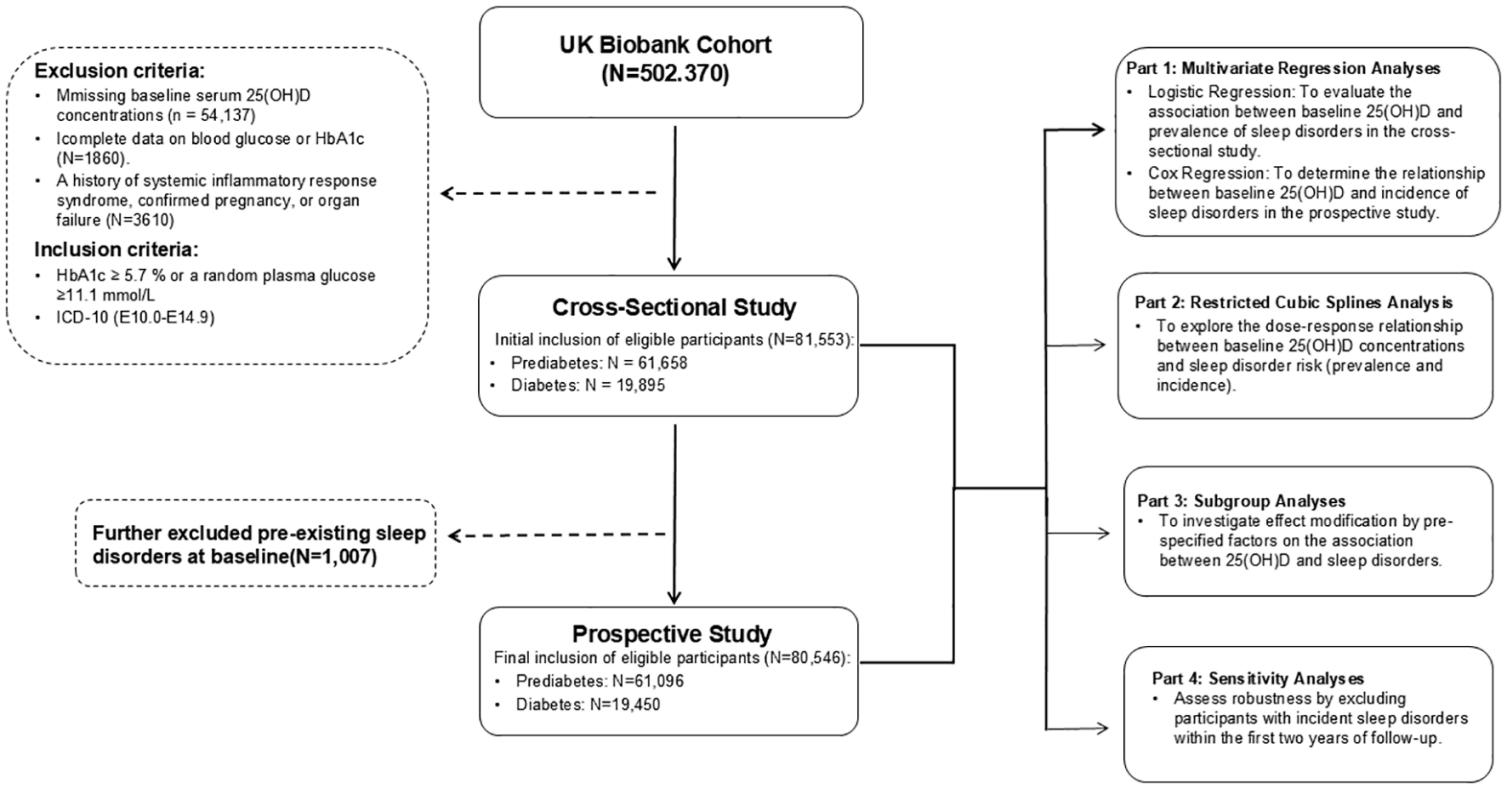
Flowchart of UK Biobank showing exclusion of participants included in the cross-sectional and prospective participants.

In this study, participants from the UK Biobank cohort were excluded based on the following criteria: missing baseline serum 25(OH)D concentrations (n = 54,137), incomplete data on blood glucose or glycated hemoglobin (HbA1c) (n = 1,860), and a history of systemic inflammatory response syndrome, pregnancy, or organ failure (n = 3,610). At baseline, eligible participants were initially included based on the American Diabetes Association (ADA) diagnostic criteria for prediabetes and diabetes mellitus, forming a cohort of81,553 individuals in a cross-sectional study (mean age 59.6 [SD 7.1] years, comprising 41,275 males [50.6%] and 40,278 females [49.4%]), comprising 61,658 individuals with prediabetes mellitus and 19,895 individuals with diabetes mellitus. For the subsequent prospective study phase, considering sleep disorders as the primary outcome of interest, we additionally excluded 1,007 participants with pre-existing sleep disorders at baseline to minimize reverse causality. The final prospective study cohort included 80,546 eligible participants (mean age 59.6 [SD 7.1] years, comprising 40,513 males [50.3%] and 40,033 females [49.7%]), consisting of 61,096 individuals with prediabetes mellitus and 19,450 individuals with diabetes mellitus at baseline. To assess the association between baseline serum 25(OH)D concentrations and the risk of sleep disorders in individuals with prediabetes mellitus and diabetes mellitus, we employed multivariate regression analyses, restricted cubic spline analysis, subgroup analyses, and sensitivity analyses. The study design is illustrated in **Figure 1**.

### 2.3 Definitions of diabetes and prediabetes

According to the American Diabetes Association (ADA) 2021 Standards of Medical Care in Diabetes (19), prediabetes was defined as a baseline HbA1c level between 5.7% and 6.4% (39–47 mmol/mol), and the definition of diabetes was based on any of the following criteria: (1) HbA1c ≥ 6.5% (48 mmol/mol) or a random plasma glucose ≥11.1 mmol/L; (2) Identification of incident diabetes events using hospital admission and diagnostic data, indicated by ICD-10 codes E10.0–E14.9.

### 2.4 Assessment of serum 25(OH)D concentrations

A series of biochemical markers, including serum 25(OH)D (nmol/L), were measured in blood samples collected at baseline (2006–2010). Serum 25(OH)D concentrations were determined using chemiluminescent immunoassay analysis on a DiaSorin Ltd. LIAISON XL platform. Calibration and quality control were conducted by UK Biobank, with a coefficient of variation for serum 25(OH)D measurement ranging from 5.04% to 6.14% and 100% accuracy in external quality assurance. The assay range was from 10 nmol/L to 375 nmol/L. Details regarding the measurement process are available on the UK Biobank website (https://biobank.ndph.ox.ac.uk/showcase/showcase/docs/serum_biochemistry.pdf).

### 2.5 Assessment of covariates

At recruitment, Sociodemographic information (age, sex, ethnicity, education, and household income), lifestyle habits (sleep, alcohol intake, and smoking status), and the season of vitamin D assessment were collected at recruitment centers in the UK Biobank. Vitamin D assessment season was classified by the month in which participants attended assessment: spring (March, April, or May), summer (June, July, or August), autumn (September, October, or November), and winter (December, January, or February). Information on medication history (use of antihypertensive drugs, lipid-lowering medications, antidiabetic drugs, and vitamin D supplements) and medical history (parathyroid disease) was also collected.

### 2.6 Outcome ascertainment

The primary outcome of this study was the presence of sleep disorders. Sleep disorder cases were identified using algorithms provided by the UK Biobank, which incorporated hospitalization and death registry data linked to health event statistics, patient records databases, Welsh patient inpatient records, and the Scottish Morbidity Records. Health outcome data were available until October 31, 2022. Sleep disorder events were defined using ICD-10 codes F51 and G47 (non-organic and organic sleep disorders), including insomnia (F51.0, G47.0), sleepwalking (F51.3), sleep apnea (G47.3), and other disorders within these categories.

### 2.7 Statistical analysis

All analyses and visualizations in this study were conducted using R version 4.2.3. For missing covariate data, we used multiple imputation by chained equations with 20 iterations.

#### 2.7.1 Baseline characteristics and follow-up

Baseline characteristics were described for the entire prospective study cohort, as well as separately for participants with prediabetes and diabetes. Participants were categorized into four groups based on baseline serum 25(OH)D concentrations, following the Endocrine Society’s Clinical Practice Guidelines: severe deficiency (<25.0 nmol/L), moderate deficiency (25.0–50.0 nmol/L), insufficiency (50.0–75.0 nmol/L), and sufficiency (≥75.0 nmol/L). Follow-up time was calculated for each participant from the date of assessment center attendance to the earliest of the following events: incident diagnosis of sleep disorder, loss to follow-up, death, or study completion (October 31, 2022). Continuous variables are presented as Mean(SD) and categorical variables as N (%). Statistical significance was defined as a two-sided p-value < 0.05.

#### 2.7.2 Multivariate regression analyses

To assess the association between baseline serum 25(OH)D concentrations and sleep disorders, multivariable regression analyses were conducted. Logistic regression analysis was employed to evaluate the cross-sectional association with prevalent sleep disorders at baseline, while Cox proportional hazards regression models were utilized to estimate the hazard ratio (HR) for incident sleep disorders during the follow-up period in the Prospective Study. Serum 25(OH)D concentration was treated as the primary exposure, and sleep disorder incidence (and prevalence at baseline) as the outcome.

Initially, serum 25(OH)D concentration was modeled as a continuous variable to estimate the effect per standard deviation (SD) increment. Subsequently, serum 25(OH)D concentration was categorized into the predefined four groups (severe deficiency, moderate deficiency, insufficiency, and sufficiency). For categorical analysis, the severe deficiency group (<25.0 nmol/L) served as the reference category, and p-values for trend were calculated to assess the monotonic dose-response relationship.

Three nested models were constructed to adjust for potential confounding. Model 1 was unadjusted. Model 2 was adjusted for core demographic and lifestyle factors: age, sex, ethnicity (White, Mixed, Asian, Black, and Other), education level (categorized), household income (categorized), season of vitamin D assessment, summer sun exposure time, and vitamin D supplement use. Model 3 further incorporated additional behavioral and clinical confounders: sleep duration (categorized), smoking status, alcohol consumption, use of antihypertensive medications, lipid-lowering medications, antidiabetic medications, and history of parathyroid disease.

#### 2.7.3 Restricted cubic splines analysis

To examine the shape of the dose-response relationship between serum 25(OH)D concentrations and the risk of sleep disorders, restricted cubic spline analysis was performed. This analysis allowed for the evaluation of potential non-linear associations on a continuous scale, visually representing the relationship across the spectrum of serum 25(OH)D concentrations.

#### 2.7.4 Subgroup analyses

Stratified analyses were conducted to explore potential effect modification by pre-specified factors on the association between serum 25(OH)D concentrations and sleep disorders. Subgroups were defined by: sex (male, female), age (≤60, >60 years), body mass index (BMI) (≤30, >30 kg/m ²), and smoking status (never, former, current, and other). Interaction terms were incorporated into regression models to formally test for statistical interaction between serum 25(OH)D concentrations and each stratified factor. When stratifying by a factor, the stratification variable itself was not included as a covariate in the model to avoid collinearity.

#### 2.7.5 Sensitivity analyses

To evaluate the robustness of the primary findings and to mitigate potential reverse causality, sensitivity analyses were performed. These analyses involved excluding participants who developed sleep disorders within the initial two years of follow-up. This approach aimed to minimize the possibility that early, subclinical sleep disorders might have influenced baseline serum 25(OH)D concentrations, rather than vice versa, thereby strengthening the inference of a forward causal relationship.

## 3 Results

### 3.1 Baseline characteristics of participants

The Prospective Study cohort comprised a total of 80,546 participants, including 61,096 individuals with prediabetes and 19,450 individuals with diabetes (**Table 1**). Among these participants at baseline, 40,513 (50.3%) were male and 40,033 (49.7%) were female. and mean (SD) age of the cohort was 59.6 (7.1). During an average follow-up of 12.8 years, 2,704 cases of sleep disorders were recorded, with 1,713 cases in the prediabetes group and 991 cases in the diabetes group. Among individuals with prediabetes and diabetes, those with sufficient serum 25(OH)D concentrations (≥ 75 nmol/L) included 5,769 (9.5%) and 1,307 (6.7%) participants, respectively, while those with severe deficiency (< 25 nmol/L) included 9,671 (15.8%) and 4,369 (22.5%) individuals, respectively. Baseline characteristics varied across different serum 25(OH)D concentration groups, particularly among the diabetic population, where participants in the sufficient serum 25(OH)D group were more likely to be older, have a lower BMI, and be White, male, daily drinkers with an average sleep duration of 7-8 hours per day. Conversely, among prediabetic individuals, those with sufficient serum 25(OH)D concentrations were more likely to be female (52.2%). Cross-Sectional Study cohort are detailed in **Supplementary Table 1**.

**Table 1:** Baseline characteristics of the prospective study participants by serum 25(OH)D.

Baseline characteristics*	Total	Serum 25(OH)D in prediabetes, nmol/L					Serum 25(OH)D in diabetes, nmol/L				
		< 25	25 to 50	50 to 75	≥ 75	P	< 25	25 to 50	50 to 75	≥ 75	P
No. of participants, n	80546	9671	26810	18846	5769		4369	8897	4877	1307	
Age, years	59.6 (7.1)	57.4 (7.7)	59.4 (7.1)	60.6 (6.5)	61.0 (6.3)	<0.001	57.7 (7.5)	59.5 (7.1)	60.8 (6.7)	61.0 (6.5)	<0.001
Sex											
Male	40513 (50.3%)	4666 (48.2%)	12499 (46.6%)	8696 (46.1%)	2759 (47.8%)	0.003	2547 (58.3%)	5452 (61.3%)	3097 (63.5%)	797 (61.0%)	<0.001
Female	40033 (49.7%)	5005 (51.8%)	14311 (53.4%)	10150 (53.9%)	301 0 (52.2%)		1822 (41.7%)	3445 (38.7%)	1780 (36.5%)	510 (39.0%)	
Body mass index, kg/m2	29.6 (5.5)	30.3 (6.0)	29.5 (5.4)	28.3 (4.7)	27.1 (4.4)	<0.001	32.4 (6.7)	31.8 (5.7)	30.5 (5.2)	28.9 (4.8)	<0.001
Ethnicity						<0.001					<0.001
White	72125 (89.5%)	7295 (75.4%)	24193 (90.2%)	18087 (96.0%)	5651 (98.0%)		3244 (74.3%)	7779 (87.4%)	4601 (94.3%)	1275 (97.6%)	
Mixed	577 (0.7%)	126 (1.3%)	195 (0.7%)	108 (0.6%)	18 (0.3%)		39 (0.9%)	64 (0.7%)	22 (0.5%)	5 (0.4%)	
Asian	3698 (4.6%)	1230 (12.7%)	958 (3.6%)	209 (1.1%)	28 (0.5%)		714 (16.3%)	443 (5.0%)	103 (2.1%)	13 (1.0%)	
Black	2548 (3.2%)	648 (6.7%)	920 (3.4%)	251 (1.3%)	39 (0.7%)		206 (4.7%)	381 (4.3%)	94 (1.9%)	9 (0.7%)	
Others	1598 (2.0%)	372 (3.8%)	544 (2.0%)	191 (1.0%)	33 (0.6%)		166 (3.8%)	230 (2.6%)	57 (1.2%)	5 (0.4%)	
Education**						<0.001					<0.001
1	20300 (25.2%)	2683 (27.7%)	7158 (26.7%)	4638 (24.6%)	1340 (23.2%)		1093 (25.0%)	2105 (23.7%)	1004 (20.6%)	279 (21.3%)	
2	27796 (34.5%)	3195 (33.0%)	9223 (34.4%)	6761 (35.9%)	2134 (37.0%)		1462 (33.5%)	2953 (33.2%)	1616 (33.1%)	452 (34.6%)	
3	11145 (13.8%)	1278 (13.2%)	3663 (13.7%)	2636 (14.0%)	801 (13.9%)		576 (13.2%)	1282 (14.4%)	714 (14.6%)	195 (14.9%)	
4	21305 (26.5%)	2515 (26.0%)	6766 (25.2%)	4811 (25.5%)	1494 (25.9%)		1238 (28.3%)	2557 (28.7%)	1543 (31.6%)	381 (29.2%)	
Household income, £						<0.001					<0.001
<18,000	21718 (27.0%)	2986 (30.9%)	6973 (26.0%)	4433 (23.5%)	1339 (23.2%)		1551 (35.5%)	2667 (30.0%)	1422 (29.2%)	347 (26.5%)	
18000-30,999	19089 (23.7%)	2055 (21.2%)	6314 (23.6%)	4728 (25.1%)	1429 (24.8%)		917 (21.0%)	2062 (23.2%)	1240 (25.4%)	344 (26.3%)	
31,000-51,999	14821 (18.4%)	1700 (17.6%)	5202 (19.4%)	3601 (19.1%)	1110 (19.2%)		679 (15.5%)	1479 (16.6%)	812 (16.6%)	238 (18.2%)	
>52,000	10970 (13.6%)	1250 (12.9%)	3803 (14.2%)	2756 (14.6%)	880 (15.3%)		443 (10.1%)	1105 (12.4%)	569 (11.7%)	164 (12.5%)	
Others	13948 (17.3%)	1680 (17.4%)	4518 (16.9%)	3328 (17.7%)	1011 (17.5%)		779 (17.8%)	1584 (17.8%)	834 (17.1%)	214 (16.4%)	
Smoking status						<0.001					<0.001
Never	38420 (47.7%)	4576 (47.3%)	13070 (48.8%)	9257 (49.1%)	2660 (46.1%)		2038 (46.6%)	4080 (45.9%)	2160 (44.3%)	579 (44.3%)	
Previous	30180 (37.5%)	2866 (29.6%)	9520 (35.5%)	7224 (38.3%)	2402 (41.6%)		1565 (35.8%)	3709 (41.7%)	2278 (46.7%)	616 (47.1%)	
Current	11479 (14.3%)	2156 (22.3%)	4099 (15.3%)	2267 (12.0%)	684 (11.9%)		735 (16.8%)	1036 (11.6%)	403 (8.3%)	99 (7.6%)	
Others	467 (0.6%)	73 (0.8%)	121 (0.5%)	98 (0.5%)	23 (0.4%)		31 (0.7%)	72 (0.8%)	36 (0.7%)	13 (1.0%)	
Drinking status						<0.001					<0.001
Never	5655 (7.0%)	1193 (12.3%)	1677 (6.3%)	825 (4.4%)	204 (3.5%)		650 (14.9%)	769 (8.6%)	290 (5.9%)	47 (3.6%)	.
Previous	4254 (5.3%)	575 (5.9%)	1302 (4.9%)	738 (3.9%)	210 (3.6%)		390 (8.9%)	648 (7.3%)	321 (6.6%)	70 (5.4%)	
Current	70452 (87.5%)	7856 (81.2%)	23777 (88.7%)	17258 (91.6%)	5348 (92.7%)		3315 (75.9%)	7455 (83.8%)	4255 (87.2%)	1188 (90.9%)	
Others	185 (0.2%)	47 (0.5%)	54 (0.2%)	25 (0.1%)	7 (0.1%)		14 (0.3%)	25 (0.3%)	11 (0.2%)	2 (0.2%)	
Sleeping duration, hours/day						<0.001					<0.001
≤6	22394 (27.8%)	3176 (32.8%)	7602 (28.4%)	4753 (25.2%)	1378 (23.9%)		1424 (32.6%)	2603 (29.3%)	1179 (24.2%)	279 (21.3%)	
7-8	50381 (62.5%)	5637 (58.3%)	16960 (63.3%)	12412 (65.9%)	3866 (67.0%)		2378 (54.4%)	5190 (58.3%)	3104 (63.6%)	834 (63.8%)	
≥9	7771 (9.6%)	858 (8.9%)	2248 (8.4%)	1681 (8.9%)	525 (9.1%)		567 (13.0%)	1104 (12.4%)	594 (12.2%)	194 (14.8%)	
Summer outdoor time, hours/day	4.1 (2.5)	3.6 (2.4)	3.9 (2.4)	4.3 (2.4)	4.6 (2.5)	<0.001	3.7 (2.5)	4.0 (2.6)	4.5 (2.5)	4.5 (2.4)	<0.001
Glucose-lowering drugs	14090 (17.5%)	391 (4.0%)	912 (3.4%)	548 (2.9%)	192 (3.3%)	<0.001	2600 (59.5%)	5430 (61.0%)	3083 (63.2%)	934 (71.5%)	<0.001
Antihypertensive drugs	18228 (22.6%)	1800 (18.6%)	4801 (17.9%)	3280 (17.4%)	1045 (18.1%)	0.083	1610 (36.9%)	3400 (38.2%)	1788 (36.7%)	504 (38.6%)	0.187
Lowering-cholesterol drugs	29744 (36.9%)	2623 (27.1%)	7257 (27.1%)	5290 (28.1%)	1915 (33.2%)	<0.001	2637 (60.4%)	5765 (64.8%)	3276 (67.2%)	981 (75.1%)	<0.001
Vitamin D assessment seasons						0.234					0.079
Spring	24125 (30.0%)	2874 (29.7%)	8111 (30.3%)	5618 (29.8%)	1716 (29.7%)		1288 (29.5%)	2649 (29.8%)	1524 (31.2%)	345 (26.4%)	
Summer	23704 (29.4%)	2773 (28.7%)	7924 (29.6%)	5578 (29.6%)	1700 (29.5%)		1313 (30.1%)	2611 (29.3%)	1389 (28.5%)	416 (31.8%)	
Fall	14436 (17.9%)	1783 (18.4%)	4645 (17.3%)	3415 (18.1%)	1034 (17.9%)		783 (17.9%)	1662 (18.7%)	872 (17.9%)	242 (18.5%)	
Winter	18281 (22.7%)	2241 (23.2%)	6130 (22.9%)	4235 (22.5%)	1319 (22.9%)		985 (22.5%)	1975 (22.2%)	1092 (22.4%)	304 (23.3%)	
Vitamin D supplements	3233 (4.0%)	176 (1.8%)	879 (3.3%)	1076 (5.7%)	438 (7.6%)	<0.001	78 (1.8%)	270 (3.0%)	243 (5.0%)	73 (5.6%)	<0.001
Parathyroid diseases	516 (0.6%)	66 (0.7%)	171 (0.6%)	85 (0.5%)	21 (0.4%)	0.004	40 (0.9%)	86 (1.0%)	35 (0.7%)	12 (0.9%)	0.516
Sleep disorder	2704 (3.4%)	361 (3.7%)	783 (2.9%)	462 (2.5%)	107 (1.9%)	<0.001	271 (6.2%)	489 (5.5%)	191 (3.9%)	40 (3.1%)	<0.001
Sleep apnea	2445 (3.0%)	327 (3.4%)	691 (2.6%)	408 (2.2%)	99 (1.7%)	<0.001	245 (5.6%)	456 (5.1%)	182 (3.7%)	37 (2.8%)	<0.001
Other sleep disorder	259 (0.3%)	34 (0.4%)	92 (0.3%)	54 (0.3%)	8 (0.1%)	0.061	26 (0.6%)	33 (0.4%)	9 (0.2%)	3 (0.2%)	0.011

*Continuous variables are presented as mean(SD), category variables are presented as N (%).

**Education:1.College or university degree;2.A/AS levels or equivalent or O levels/GCSE or CSE or equivalent;3.NVQ or HND or HNC or equivalent or other professional qualifications;4.others.

### 3.2 Cross-sectional study

#### 3.2.1 Logistic regression analysis in the cross-sectional participants

Cross-sectional study revealed a significant association between serum 25(OH)D concentrations and sleep disorder risk (**Table 2**). In the fully adjusted Model 3, compared to the severely deficient serum 25(OH)D group (< 25 nmol/L), the odds of sleep disorders in the prediabetic population were approximately 28% lower in the deficient group (25–50 nmol/L) (OR = 0.72; 95% CI: 0.58–0.91), 32% lower in the insufficient group (50–75 nmol/L) (OR = 0.68; 95% CI: 0.53–0.87), and 47% lower in the sufficient group (≥ 75 nmol/L) (OR = 0.53; 95% CI: 0.36–0.77). For each standard deviation (SD) increase in serum 25(OH)D concentrations, there was an average 16% reduction in the risk of sleep disorders (OR = 0.84; 95% CI: 0.77–0.92, p < 0.001), indicating a dose-response relationship.

**Table 2:** Odds ratios (95% confidence intervals) of sleep disorders based on serum 25(OH)D concentrations in individuals with prediabetes and diabetes.

Serum 25 (OH)D	Event/Total (%)	Model 1			Model 2			Model 3		
		OR (95% CI)	P overall	P for trend	OR (95% CI)	P overall	P for trend	OR (95% CI)	P overall	P for trend
Prediabetes (n= 61658)										
< 25	123/9794(1.26%)	Reference		<0.001	Reference		<0.001	Reference		<0.001
25 to 50	243/27053(0.90%)	0.71 (0.57-0.89)	0.002		0.70 (0.56-0.88)	0.002		0.72 (0.58-0.91)	0.005	
50 to 75	158/19004(0.83%)	0.66 (0.52-0.84)	0.001		0.65 (0.51-0.84)	0.001		0.68 (0.53-0.87)	0.003	
≥ 75	38/5807(0.65%)	0.52 (0.36-0.75)	<0.001		0.51 (0.35-0.74)	<0.001		0.53 (0.36-0.77)	0.001	
Per SD	562/61658(0.91%)	0.83 (0.76-0.91)	<0.001		0.83 (0.76-0.91)	<0.001		0.84 (0.77-0.92)	<0.001	
Diabetes (n=19895)										
< 25	116/4485(2.59%)	Reference		0.005	Reference		0.002	Reference		0.016
25 to 50	210/9107(2.31%)	0.89 (0.71-1.12)	0.315		0.86 (0.68-1.09)	0.208		0.89 (0.70-1.13)	0.329	
50 to 75	101/4978(2.03%)	0.78 (0.60-1.02)	0.071		0.73 (0.55-0.97)	0.032		0.80 (0.61-1.07)	0.134	
≥ 75	18/1325(1.36%)	0.52 (0.31-0.86)	0.010		0.50 (0.30-0.83)	0.008		0.54 (0.32-0.90)	0.019	
Per SD	445/19895(2.24%)	0.84 (0.76-0.93)	0.001		0.82 (0.74-0.91)	<0.001		0.85 (0.76-0.94)	0.002	

Model 1: unadjusted model.

Model 2: adjusted for age, sex (male, female), ethnicity (White, Mixed, Asian, Black, Others), education (college or university degree, A/AS levels or equivalent or O levels/GCSEs, NVQ or HND or HNC or equivalent or other professional qualifications, Others), house income(£18,000, £18,000-30,999,£31,000-51,999,> £52,000, Others), summer outdoor time (hours/day),vitamin D assessment season (spring, summer, fall, or winter), vitamin D supplements(yes or no).

Model 3: adjusted for model 2 variables as well as sleep duration (≤6, 7-8, ≥9 hours/day), Smoking status (Never, Previous, Current, Others), Drinking status (Never, Previous, Current, Others), Glucose-lowering drugs (yes, no). Antihypertensive drugs (yes, no), Lowering-cholesterol drugs (yes, no), Parathyroid diseases (yes, no).

The association remained significant in unadjusted Model 1 and partially adjusted Model 2. In these models, individuals in the prediabetes group with sufficient serum 25(OH)D concentrations exhibited a notably lower risk of sleep disorders compared to those with severe deficiency (Model 1: OR = 0.52; 95% CI: 0.36–0.75; Model 2: OR = 0.51; 95% I: 0.35–0.74). A significant negative trend (p for trend < 0.001) between serum 25(OH)D concentrations and sleep disorder risk was observed in the prediabetes group. A similar trend was identified in the diabetes group, where higher serum 25(OH)D concentrations correlated with reduced sleep disorder risk (p for trend < 0.05).

In addition, the results of the Restricted Cubic Spline Analysis and Subgroup Analysis from the cross-sectional study have been included in the **Supplementary Materials** (**Supplementary Figures 1**-**3**).

### 3.3 Prospective study

#### 3.3.1 Cox regression analysis in the prospective participants

During the mean follow-up period of 12.8 years, we documented 1,713 sleep disorder cases among individuals with prediabetes and 991 cases among those with diabetes. Our analysis confirmed a significant impact of serum 25(OH)D concentrations on the incidence of sleep disorders in both the prediabetes and diabetes groups (**Table 3**).

**Table 3:** Hazard ratios (95% confidence intervals) of sleep disorders based on serum 25(OH)D concentrations in individuals with prediabetes and diabetes.

Serum 25 (OH)D	Event/Total (%)	Model 1			Model 2			Model 3		
		HR (95% CI)	P overall	P for trend	HR (95% CI)	P overall	P for trend	HR (95% CI)	P overall	P for trend
Prediabetes (n= 61096)										
< 25	361/9671(3.73%)	Reference		<0.001	Reference		<0.001	Reference		<0.001
25 to 50	783/26810(2.92%)	0.77 (0.68-0.87)	<0.001		0.78 (0.69-0.89)	<0.001		0.80 (0.70-0.91)	<0.001	
50 to 75	462/18846(2.45%)	0.64 (0.56-0.74)	<0.001		0.66 (0.57-0.76)	<0.001		0.69 (0.60-0.80)	<0.001	
≥ 75	107/5769(1.85%)	0.49 (0.39-0.60)	<0.001		0.50 (0.40-0.62)	<0.001		0.52 (0.41-0.65)	<0.001	
Per SD	1713/61096(2.80%)	0.81 (0.77-0.86)	<0.001		0.82 (0.78-0.86)	<0.001		0.83 (0.79-0.88)	<0.001	
Diabetes (n=19450)										
< 25	271/4369(6.20%)	Reference		<0.001	Reference		<0.001	Reference		<0.001
25 to 50	489/8897(5.50%)	0.86 (0.74-1.00)	0.050		0.83 (0.71-0.97)	0.017		0.84 (0.72-0.98)	0.029	
50 to 75	191/4877(3.92%)	0.60 (0.50-0.73)	<0.001		0.57 (0.47-0.70)	<0.001		0.60 (0.50-0.73)	<0.001	
≥ 75	40/1307(3.06%)	0.48 (0.34-0.66)	<0.001		0.47 (0.33-0.65)	<0.001		0.48 (0.34-0.67)	<0.001	
Per SD	991/19450(5.10%)	0.81 (0.76-0.87)	<0.001		0.80 (0.74-0.86)	<0.001		0.81 (0.76-0.87)	<0.001	

Model 1: unadjusted model.

Model 2: adjusted for age, sex (male, female), ethnicity (White, Mixed, Asian, Black, Others), education (college or university degree, A/AS levels or equivalent or O levels/GCSEs, NVQ or HND or HNC or equivalent or other professional qualifications, Others), house income(£18,000, £18,000-30,999,£31,000-51,999,> £52,000, Others), summer outdoor time (hours/day),vitamin D assessment season (spring, summer, fall, or winter), vitamin D supplements(yes or no).

Model 3: adjusted for model 2 variables as well as sleep duration (≤6, 7-8, ≥9 hours/day), Smoking status (Never, Previous, Current, Others), Drinking status (Never, Previous, Current, Others), Glucose-lowering drugs (yes, no). Antihypertensive drugs (yes, no), Lowering-cholesterol drugs (yes, no), Parathyroid diseases (yes, no).

In Model 1, with no adjustments, compared to the severely deficient group, the risk of sleep disorders decreased by 36% (HR = 0.64; 95% CI: 0.56–0.74) and 51% (HR = 0.49; 95% CI: 0.39–0.60) in the insufficient and sufficient groups of the prediabetes cohort, respectively. In the diabetes cohort, these risks were reduced by 40% (HR = 0.60; 95% CI: 0.50–0.73) and 52% (HR = 0.48; 95% CI: 0.34–0.66), respectively.

After partial adjustment in Model 2, the negative correlation between serum 25(OH)D concentrations and sleep disorder risk remained significant (p for trend <0.001). For each SD increase in serum 25(OH)D concentration, sleep disorder risk was reduced by 18% in the prediabetes group (HR = 0.82; 95% CI: 0.78–0.86) and 20% in the diabetes group (HR = 0.80; 95% CI: 0.74–0.86).

In the fully adjusted Model 3, an even more pronounced trend was observed, with a gradual decline in sleep disorder risk associated with increasing serum 25(OH)D concentrations (p for trend < 0.001). Compared to the severely deficient group, hazard ratios (HRs) and 95% confidence intervals (CIs) for sleep disorders in the prediabetes group were 0.80 (95% CI: 0.70–0.91) for moderate deficiency, 0.69 (95% CI: 0.60–0.80) for insufficiency, and 0.52 (95% CI: 0.41–0.65) for sufficiency. In the diabetes group, these HRs were 0.84 (95% CI: 0.72–0.98), 0.60 (95% CI: 0.50–0.73), and 0.48 (95% CI: 0.34–0.67), respectively.

#### 3.3.2 Restricted cubic spline analysis

**Figure 2:**
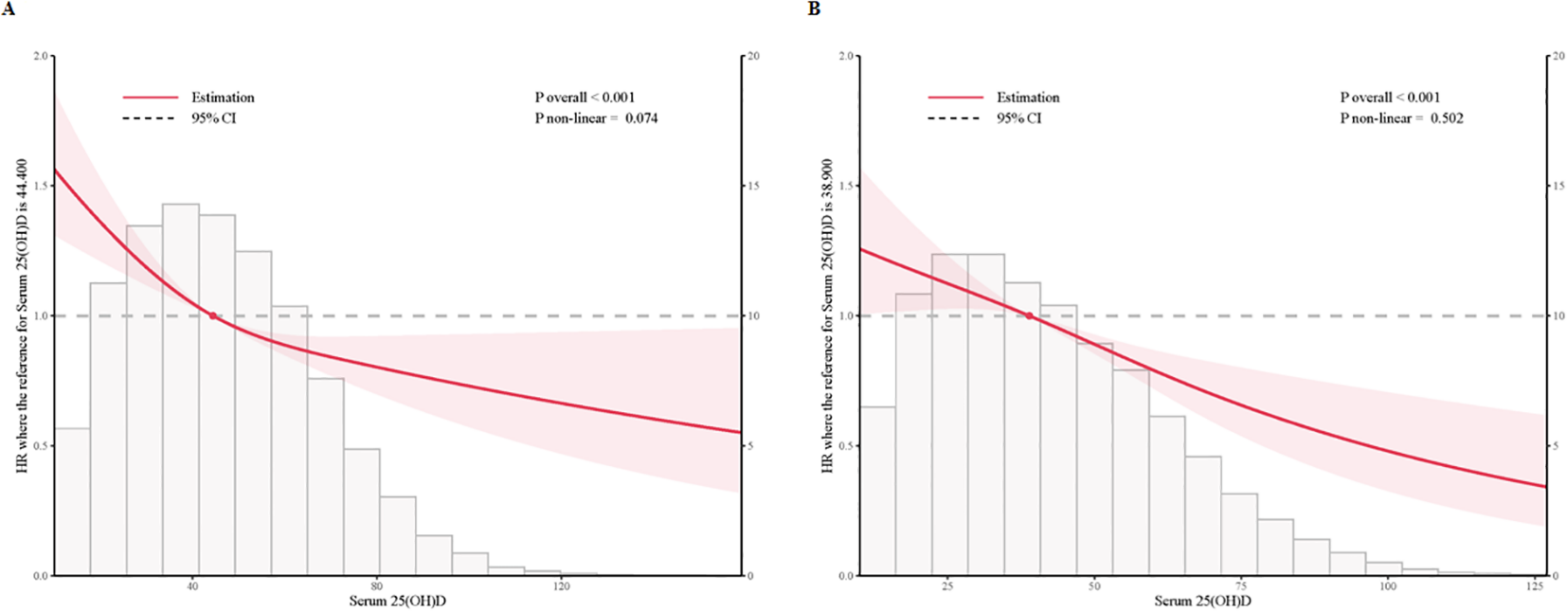
Multivariable adjusted restricted cubic splines of the hazard ratios of sleep disorders based on serum 25(OH)D concentrations in individuals with prediabetes and diabetes. **(A)** Multivariable adjusted restricted cubic splines of the hazard ratios of sleep disorders based on serum 25(OH)D concentrations in individuals with prediabetes. **(B)** Multivariable adjusted restricted cubic splines of the hazard ratios of sleep disorders based on serum 25(OH)D concentrations in individuals with diabetes.

To examine dose-response relationships, we applied fully adjusted restricted cubic spline models. The analysis found no statistically significant evidence of non-linearity (p > 0.05), supporting a linear association. Both cross-sectional and prospective analyses demonstrated a linear, inverse relationship between serum 25(OH)D levels and sleep disorder risk in individuals with prediabetes and diabetes, indicating that higher serum 25(OH)D levels are consistently associated with a lower risk of sleep disorders (**Figure 2**, **Supplementary Figure 1**).

#### 3.3.3 Subgroup analysis

We conducted subgroup analyses by age (≤60, >60 years), sex (male, female), BMI (<30, ≥30 kg/m²), and smoking status (never, former, current, others).

**Figure 3:**
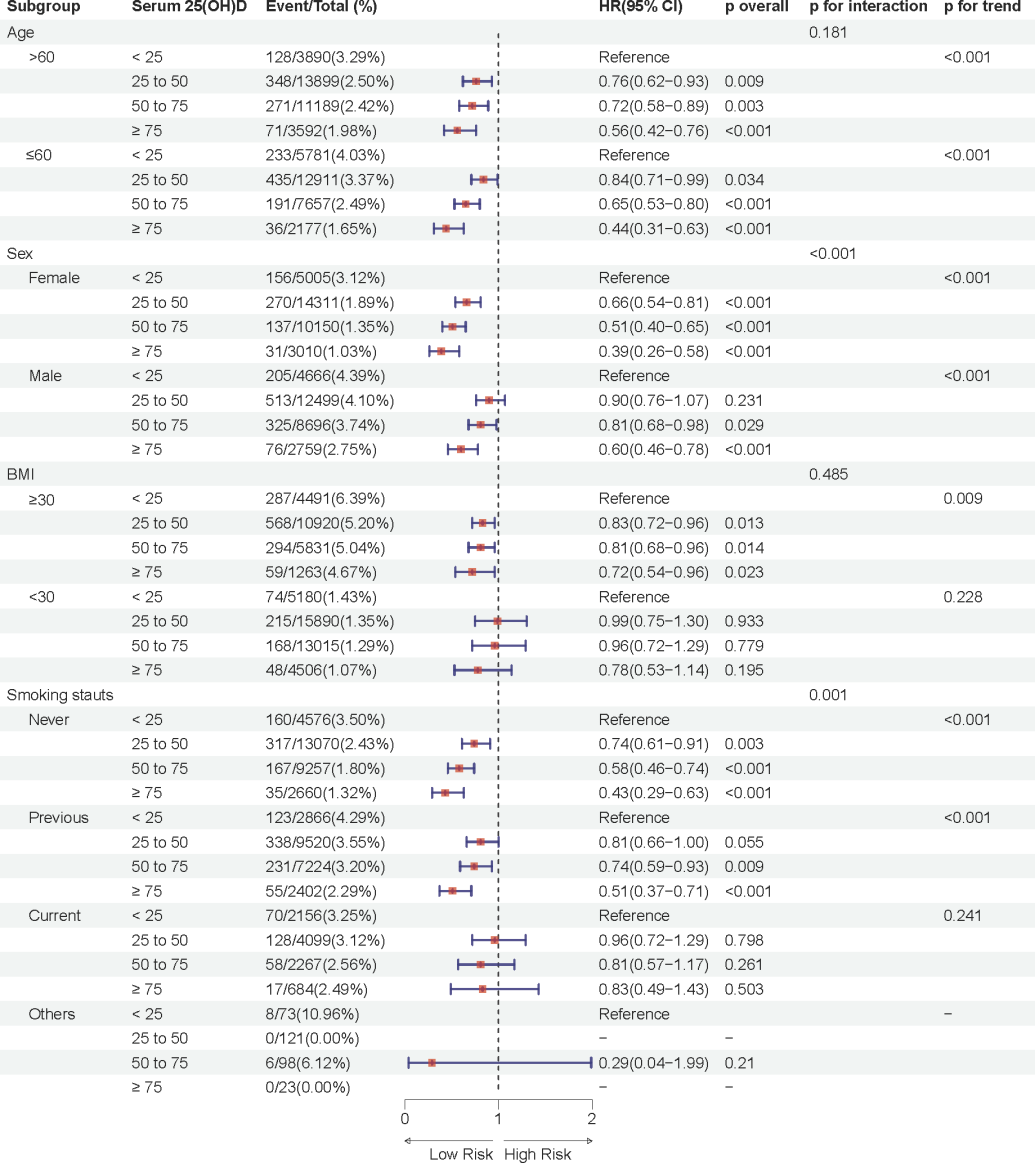
Multivariable adjusted hazard ratios (95% confidence intervals) of sleep disorders based on serum 25(OH)D concentrations in individuals with prediabetes, stratified by age, sex, body mass index (BMI), and smoking status.

**Figure 4:**
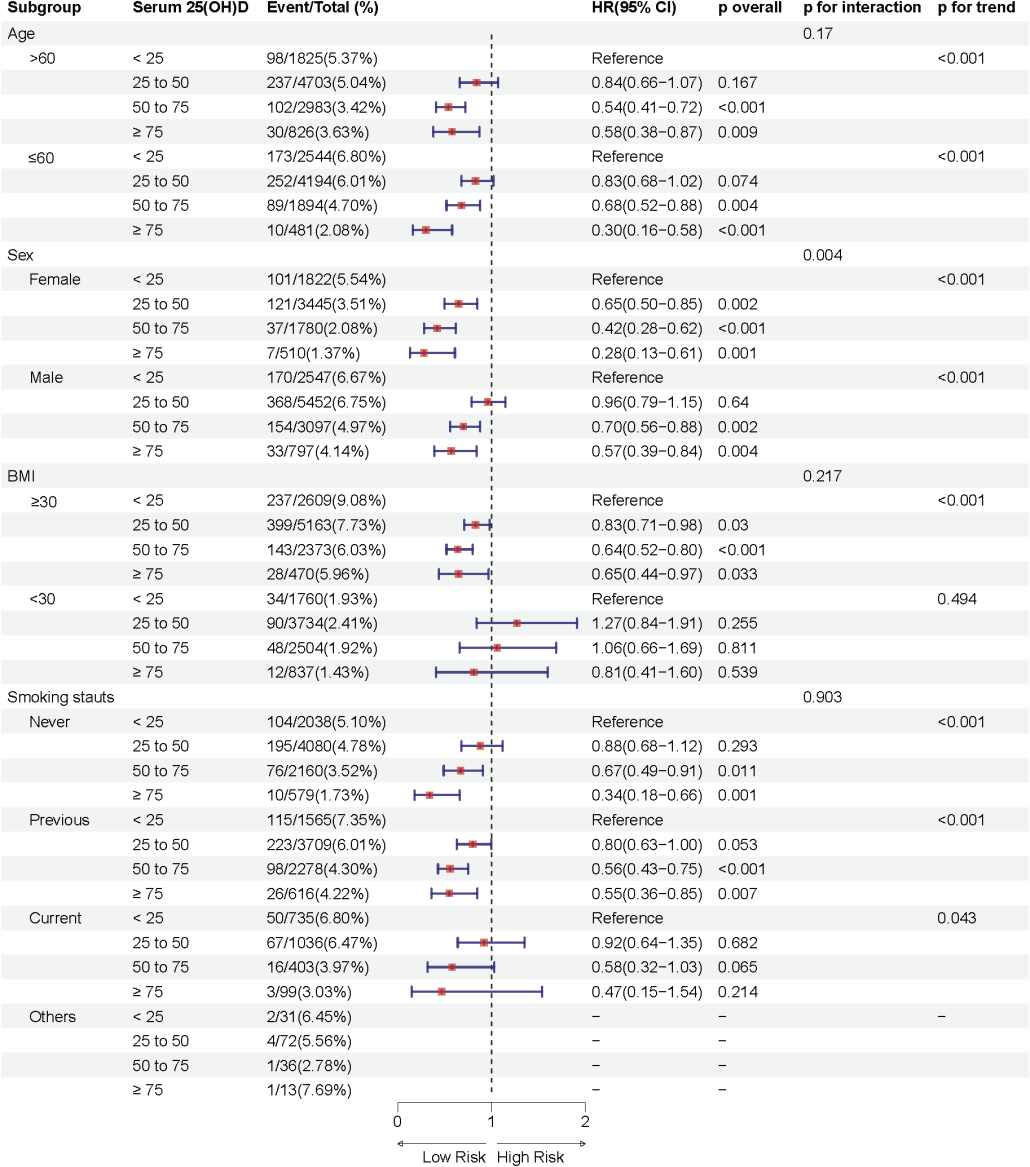
Multivariable adjusted hazard ratios (95% confidence intervals) of sleep disorders based on serum 25(OH)D concentrations in individuals with diabetes, stratified by age, sex, body mass index (BMI), and smoking status.

Notably, the association between serum 25(OH)D concentration and sleep disorder risk was more pronounced in females across both prediabetes and diabetes groups (P interaction < 0.05), suggesting potential sex-related differences in the effect of serum 25(OH)D on sleep health. In the prediabetes group, female participants with moderate deficiency, insufficiency, and sufficiency in serum 25(OH)D concentrations had approximately 34%, 49%, and 61% lower risks of sleep disorders, respectively, compared to the severely deficient group, while the respective reductions in male participants were 10%, 21%, and 40% (**Figure 3**). Similarly, in the diabetes group, females in these groups showed a 35%, 58%, and 72% lower risk of sleep disorders, while males showed reductions of 4%, 30%, and 43% (**Figure 4**). These results suggest that the relationship between serum 25(OH)D and sleep disorders varies by sex, with a stronger association observed in females with abnormal glucose metabolism.

Additional analyses indicated a significant interaction effect between smoking status and serum 25(OH)D concentrations on sleep disorder risk in the prediabetes group (P interaction = 0.001), with never-smokers having a substantially lower risk of sleep disorders compared to former smokers. Moreover, significant differences in sleep disorder risk were observed in subgroups with age ≤ 60 years and BMI ≥30 kg/m^2^ across different serum 25(OH)D concentrations (p < 0.05).

Furthermore, we conducted stratified analyses based on sleep disorder types (sleep apnea, other sleep disorders). The results showed that the association between serum 25(OH)D levels and sleep disorder risk was more pronounced in individuals with sleep apnea, with a stronger inverse relationship observed in both prediabetes and diabetes groups. In contrast, the association with other sleep disorders was weaker but still statistically significant. These findings further confirm that the impact of serum 25(OH)D levels on sleep health may vary by sleep disorder type, with a more substantial effect observed in sleep apnea cases. (**Supplementary Tables 2**, **3**).

#### 3.3.4 Sensitivity analysis

To further corroborate our findings, we conducted sensitivity analyses by excluding participants who developed sleep disorders within the initial two years of follow-up. Additional adjustments were made for potential confounding variables, and trend tests were performed to assess robustness. Results from these sensitivity analyses were consistent with the primary findings, reinforcing the association between serum 25(OH)D concentrations and sleep disorder risk in both prediabetic and diabetic populations.

In the fully adjusted model, participants in the sufficient serum 25(OH)D group (≥ 75 nmol/L) demonstrated a notably lower risk of sleep disorders, with hazard ratios (HRs) of 0.56 (95% CI: 0.44–0.70) in the prediabetes group and 0.47 (95% CI: 0.33–0.68) in the diabetes group, when compared to those in the severely deficient group (< 25 nmol/L). Moreover, each standard deviation (SD) increase in serum 25(OH)D concentration was associated with an approximate 15% reduction in sleep disorder risk (HR = 0.85; 95% CI: 0.80–0.90) in the prediabetes group and a 17% reduction (HR = 0.83; 95% CI: 0.77–0.90) in the diabetes group (**Supplementary Table 4**).

These findings confirm the association between higher serum 25(OH)D concentrations and a lower risk of sleep disorders, indicating that the observed effects are consistent and unlikely to be driven by short-term variations or early incident cases of sleep disorders.

## 4 Discussion

This study, employing both cross-sectional and prospective cohort designs, systematically investigates the association between serum 25(OH)D levels and the risk of sleep disorders in individuals with prediabetes and diabetes. First, the cross-sectional study revealed a significant inverse association between serum 25(OH)D levels and the risk of sleep disorders. Building on this, our prospective study further confirmed this crucial finding. Over an average follow-up period of 12.8 years, we observed that individuals with sufficient serum 25(OH)D levels (≥75 nmol/L) had a significantly lower risk of developing sleep disorders compared to those with severe vitamin D deficiency. Specifically, the risk was reduced by 48% in the prediabetes group and by 52% in the diabetes group. These findings suggest that maintaining adequate vitamin D levels may help reduce the prevalence and future risk of sleep disorders in individuals with impaired glucose metabolism. Moreover, subgroup analyses indicated that this association was more pronounced in specific populations, including individuals aged ≤60 years, non-smokers, those with a BMI ≥30 kg/m², and females.

This study is the first to explore the association between serum 25(OH)D concentrations and the risk of sleep disorders in individuals with dysglycemia, including those with prediabetes and diabetes. Our findings are generally consistent with previous research on the relationship between vitamin D and sleep disorders. Increasing evidence suggests that vitamin D deficiency is associated with poor sleep quality (20), reduced sleep duration (21), and increased sleep disorder risk (13), with similar associations observed in various populations, such as the elderly (22), community dwellers (23), and individuals with obesity (24). An 8-week randomized controlled trial (25) found that high-dose vitamin D supplementation alleviated fatigue, reduced anxiety, and improved cognitive function. However, it is important to note that not all studies support the positive effects of vitamin D on sleep. For instance, a randomized controlled trial in overweight or obese postmenopausal women (26) found that daily supplementation with 2000 IU of vitamin D for 12 months did not improve sleep quality, depressive symptoms, or health-related quality of life. This result contrasts with our findings and those of most other studies, suggesting that the relationship between vitamin D and sleep may not be linear or unidirectional, and could be influenced by factors such as population characteristics, vitamin D supplementation dosage, and study design.

Sleep is a complex physiological process finely regulated by various factors, including neurotransmitters (27), hormones (28), inflammatory status (29), and circadian rhythms (30). Individuals with dysglycemia are at a higher risk for sleep disorders (31). In addition to hyperglycemia caused by insulin resistance, which may affect neurotransmitter levels in the brain related to sleep regulation (32, 33), the common vitamin D deficiency in these individuals may also play a significant role (34). Some studies suggest (35, 36) that low vitamin D levels may indirectly impact sleep quality and duration by contributing to conditions such as chronic pain, myopathy, cardiovascular diseases, and mood disorders. Vitamin D, a multifunctional hormone, has receptors widely distributed in various tissues (37), including the brain. Research indicates that vitamin D may influence sleep health through several mechanisms, with the inflammatory pathway being widely recognized (38, 39). A meta-analysis demonstrated (40) elevated pro-inflammatory cytokines (such as TNF-α, IL-8, and IL-1β) in OSA patients compared to controls. Additionally, a study in the United States highlighted the essential role of IL-6 in sleep regulation (29). Although these studies suggest that vitamin D may influence sleep quality through its modulation of inflammatory responses (36), the precise mechanisms by which vitamin D affects sleep still require further investigation.

The strength of this study lies in its combination of cross-sectional and prospective designs, which provides a comprehensive analysis of the relationship between serum 25(OH)D concentrations and the risk of sleep disorders. The cross-sectional study allowed us to assess participants’ vitamin D levels and sleep disorder status at a specific point in time, enabling the identification of potential associations. The prospective study, with long-term follow-up, allowed us to observe the impact of serum 25(OH)D concentrations on the incidence of sleep disorders over time. This combined approach not only enhances the reliability of the results but also provides stronger support for causal inference (41).

Another strength of this study is the large sample size and focus on individuals with dysglycemia, which further increases the reliability and novelty of the findings. Our study found that the effect of vitamin D supplementation on reducing sleep disorder risk was most pronounced in individuals aged ≤60 years and in females. These findings suggest that age and sex may be important factors influencing the development of sleep disorders, and may also be related to variations in vitamin D concentrations among different subgroups. For example, studies have indicated that the prevalence of obstructive sleep apnea (OSA) is higher among women, particularly postmenopausal women (42). This may be due to hormonal changes that make women more susceptible to sleep problems, such as reduced sleep quality, sleep deprivation, and increased risks of OSA, restless legs syndrome, and insomnia during menopause (43–45). Recent clinical research has further elucidated the nuanced relationship between vitamin D and sex (46). Specifically, the inverse correlation between serum vitamin D and insulin resistance was statistically significant only in women with vitamin D deficiency, and not in men or women with sufficient vitamin D levels. This suggests a potential threshold effect and underscores the importance of considering sex as a critical biological factor in vitamin D’s impact on metabolic health, potentially extending to sleep regulation. In addition to age and sex, our subgroup analysis also found that BMI and smoking status may modulate the effects of vitamin D on sleep disorder risk. These results underscore the importance of considering individual characteristics when designing personalized interventions for sleep disorders.

However, this study has several limitations that should be acknowledged. First, although our findings suggest an association between higher vitamin D concentrations and reduced sleep disorder risk, as an observational study, we cannot fully exclude the influence of residual confounding factors, and causality cannot be determined. Depression is a potential confounding factor that affects a significant proportion of individuals with diabetes (47). Depression often leads to poor social interactions, dietary habits, and obesity, all of which can impact vitamin D levels (48). Furthermore, the impact of hormones on vitamin D metabolism, particularly in women (49), including menopausal status and the use of vitamin D supplements, requires further exploration.

Second, the reliance on large biobank and electronic medical records introduces potential limitations. Specifically, we used ICD codes for sleep disorders, which encompass a wide range of conditions such as insomnia, hypersomnia, narcolepsy, circadian rhythm disorders, and restless legs syndrome. The lack of more specific diagnoses, such as obstructive sleep apnea (OSA), which is particularly common in individuals with diabetes, may limit the accuracy of our findings. Future studies with more detailed sleep disorder classifications would enhance the validity of these associations. In addition, vitamin D levels were measured only at baseline, limiting our ability to capture changes over time or assess the impact of lifestyle modifications. Moreover, due to the limitations of the database, we did not include fasting blood glucose measurements, which would have provided better insight into the relationship between blood sugar control and sleep disorders. Finally, the storage and viability of serum vitamin D samples over time were not specified, which may impact the accuracy of the measurements.

## 5 Conclusion

This study contributes new insights into the preventive potential of vitamin D supplementation for sleep disorders in individuals with abnormal glucose metabolism. Future research, particularly randomized controlled trials, should investigate the therapeutic efficacy of vitamin D in improving sleep quality and reducing sleep disorder incidence. Additionally, understanding the biological mechanisms linking vitamin D to sleep regulation and examining potential interactions with other risk factors may facilitate the development of targeted, personalized interventions to optimize sleep health and quality of life for at-risk populations.

## Data Availability

The datasets presented in this study can be found in online repositories, and the data was accessed under Application Number 89483. The names of the repository/repositories can be found below: http://www.ukbiobank.ac.uk/.

## References

[1] Stranges S Tigbe W Gomez-Olive FX Thorogood M Kandala NB. Sleep problems: an emerging global epidemic? findings from the INDEPTH WHO-SAGE study among more than 40,000 older adults from 8 countries across africa and asia. Sleep. (2012) 35:1173–81. doi: 10.5665/sleep.2012 PMC3397790 22851813

[2] Sateia MJ. International classification of sleep disorders-third edition: highlights and modifications. Chest. (2014) 146:1387–94. doi: 10.1378/chest.14-0970 25367475

[3] Ohayon MM. Epidemiology of insomnia: what we know and what we still need to learn. Sleep Med Rev. (2002) 6:97–111. doi: 10.1053/smrv.2002.0186 12531146

[4] Hossein-nezhad A Holick MF. Vitamin D for health: a global perspective. Mayo Clin Proc. (2013) 88:720–55. doi: 10.1016/j.mayocp.2013.05.011 PMC3761874 23790560

[5] Lips P. Vitamin D physiology. Prog Biophys Mol Biol. (2006) 92:4–8. doi: 10.1016/j.pbiomolbio.2006.02.016 16563471

[6] Chen X Xu J Wan Z Geng T Zhu K Li R. Vitamin D and heart failure risk among individuals with type 2 diabetes: observational and mendelian randomization studies. Am J Clin Nutr. (2024) 120:491–98. doi: 10.1016/j.ajcnut.2024.07.019 39053885

[7] Harrison SR Li D Jeffery LE Raza K Hewison M. Vitamin d, autoimmune disease and rheumatoid arthritis. Calcif Tissue Int. (2020) 106:58–75. doi: 10.1007/s00223-019-00577-2 31286174 PMC6960236

[8] Latic N Erben RG. Vitamin D and cardiovascular disease, with emphasis on hypertension, atherosclerosis, and heart failure. Int J Mol Sci. (2020) 21:6483. doi: 10.3390/ijms21186483 32899880 PMC7555466

[9] Munoz A Grant WB. Vitamin D and cancer: An historical overview of the epidemiology and mechanisms. Nutrients. (2022) 14:1448. doi: 10.3390/nu14071448 35406059 PMC9003337

[10] Schiza S Bouloukaki I Kaditis A Lombardi C Bonsignore MR. Vitamin D deficiency: A forgotten aspect in sleep disorders? a critical update. Sleep Med. (2024) 121:77–84. doi: 10.1016/j.sleep.2024.06.023 38941960

[11] Casseb G Kaster MP Rodrigues A. Potential role of vitamin D for the management of depression and anxiety. CNS Drugs. (2019) 33:619–37. doi: 10.1007/s40263-019-00640-4 31093951

[12] Jiang J Tan H Xia Z Li J Zhou S Huang T. Serum vitamin D concentrations and sleep disorders: insights from NHANES 2011-2016 and mendelian randomization analysis. Sleep Breath. (2024) 28:1679–90. doi: 10.1007/s11325-024-03031-2 PMC11303418 38739211

[13] Upala S Sanguankeo A. Association between 25-hydroxyvitamin D and obstructive sleep apnea: A systematic review and meta-analysis. J Clin Sleep Med. (2015) 11:1347. doi: 10.5664/jcsm.5208 26235155 PMC4623137

[14] Harding JL Pavkov ME Magliano DJ Shaw JE Gregg EW. Global trends in diabetes complications: a review of current evidence. Diabetologia. (2019) 62:3–16. doi: 10.1007/s00125-018-4711-2 30171279

[15] McCarthy K Laird E O'Halloran AM Walsh C Healy M Fitzpatrick AL. Association between vitamin D deficiency and the risk of prevalent type 2 diabetes and incident prediabetes: A prospective cohort study using data from the irish longitudinal study on ageing (TILDA). EClinicalMedicine. (2022) 53:101654. doi: 10.1016/j.eclinm.2022.101654 36147626 PMC9486023

[16] Abrahao GP Santos MC Vieira FJ Dal Fabbro AL Franco LJ Moises RS. Serum 25-hydroxyvitamin D concentration and its association with glucose intolerance in an indigenous population. Clin Nutr. (2021) 40:1318–22. doi: 10.1016/j.clnu.2020.08.015 32900517

[17] Isaia G Giorgino R Adami S. High prevalence of hypovitaminosis D in female type 2 diabetic population. Diabetes Care. (2001) 24:1496. doi: 10.2337/diacare.24.8.1496 11473093

[18] Al AT Ashfaq A Saheb SN Abusnana S Mussa BM. Investigating the association between diabetic neuropathy and vitamin D in emirati patients with type 2 diabetes mellitus. Cells. (2023) 12:198. doi: 10.3390/cells12010198 36611991 PMC9818413

[19] Sudlow C Gallacher J Allen N Beral V Burton P Danesh J. UK biobank: an open access resource for identifying the causes of a wide range of complex diseases of middle and old age. PloS Med. (2015) 12:e1001779. doi: 10.1371/journal.pmed.1001779 25826379 PMC4380465

[20] Singh AK Kumar S Mishra S Rajotiya S Debnath S Raj P. The effects of vitamin D levels on physical, mental health, and sleep quality in adults: a comprehensive investigation. Front Nutr. (2024) 11:1451037. doi: 10.3389/fnut.2024.1451037 39619283 PMC11604434

[21] Bertisch SM Sillau S de Boer IH Szklo M Redline S. 25-hydroxyvitamin D concentration and sleep duration and continuity: Multi-ethnic study of atherosclerosis. Sleep. (2015) 38:1305–11. doi: 10.5665/sleep.4914 PMC4507736 25669179

[22] Goswami U Ensrud KE Paudel ML Redline S Schernhammer ES Shikany JM. Vitamin D concentrations and obstructive sleep apnea in a multicenter cohort of older males. Ann Am Thorac Soc. (2016) 13:712–18. doi: 10.1513/AnnalsATS.201507-440OC PMC5018890 26845389

[23] Knutson KL Wu D Patel SR Loredo JS Redline S Cai J. Association between sleep timing, obesity, diabetes: The hispanic community health Study/Study of latinos (HCHS/SOL) cohort study. Sleep. (2017) 40:zsx014. doi: 10.1093/sleep/zsx014 28329091 PMC6410944

[24] Zhang Y Zhang Y Ye Z Zhou C Yang S Liu M. Relationship of serum 25-hydroxyvitamin d, obesity with new-onset obstructive sleep apnea. Int J Obes (Lond). (2024) 48:218–23. doi: 10.1038/s41366-023-01402-5 37891401

[25] Charoenporn V Tungsukruthai P Teacharushatakit P Hanvivattanakul S Sriyakul K Sukprasert S. Effects of an 8-week high-dose vitamin D supplementation on fatigue and neuropsychiatric manifestations in post-COVID syndrome: A randomized controlled trial. Psychiatry Clin Neurosci. (2024) 78:595–604. doi: 10.1111/pcn.13716 39072958

[26] Mason C de Dieu TJ Duggan C Wang CY Korde L McTiernan A. Repletion of vitamin D associated with deterioration of sleep quality among postmenopausal women. Prev Med. (2016) 93:166–70. doi: 10.1016/j.ypmed.2016.09.035 PMC5118122 27687537

[27] Leslie M. How a neurotransmitter drives brainwashing during sleep. Science. (2025) 387:127. doi: 10.1126/science.adv7965 39787213

[28] Morssinkhof M van der Werf YD van den Heuvel OA van den Ende DA van der Tuuk K den Heijer M. Influence of sex hormone use on sleep architecture in a transgender cohort. Sleep. (2023) 46:zsad249. doi: 10.1093/sleep/zsad249 37715990 PMC10636253

[29] Vgontzas AN Zoumakis E Lin HM Bixler EO Trakada G Chrousos GP. Marked decrease in sleepiness in patients with sleep apnea by etanercept, a tumor necrosis factor-alpha antagonist. J Clin Endocrinol Metab. (2004) 89:4409–13. doi: 10.1210/jc.2003-031929 15356039

[30] Lok R Qian J Chellappa SL. Sex differences in sleep, circadian rhythms, and metabolism: Implications for precision medicine. Sleep Med Rev. (2024) 75:101926. doi: 10.1016/j.smrv.2024.101926 38564856

[31] Subramanian A Adderley NJ Tracy A Taverner T Hanif W Toulis KA. Risk of incident obstructive sleep apnea among patients with type 2 diabetes. Diabetes Care. (2019) 42:954–63. doi: 10.2337/dc18-2004 30862657

[32] Ficker JH Dertinger SH Siegfried W Konig HJ Pentz M Sailer D. Obstructive sleep apnoea and diabetes mellitus: the role of cardiovascular autonomic neuropathy. Eur Respir J. (1998) 11:14–9. doi: 10.1183/09031936.98.11010014 9543264

[33] Khalil M Power N Graham E Deschenes SS Schmitz N. The association between sleep and diabetes outcomes - a systematic review. Diabetes Res Clin Pract. (2020) 161:108035. doi: 10.1016/j.diabres.2020.108035 32006640

[34] Wang M Zhou T Li X Ma H Liang Z Fonseca VA. Baseline vitamin D status, sleep patterns, and the risk of incident type 2 diabetes in data from the UK biobank study. Diabetes Care. (2020) 43:2776–84. doi: 10.2337/dc20-1109 PMC7576418 32847829

[35] Nikolac GN Unic A Miler M Pavicic T Culej J Bolanca I. In sickness and in health: pivotal role of vitamin d. Biochem Med (Zagreb). (2020) 30:20501. doi: 10.11613/BM.2020.020501 PMC7271749 32550812

[36] Grant WB Wimalawansa SJ Pludowski P Cheng RZ. Vitamin D: Evidence-based health benefits and recommendations for population guidelines. Nutrients. (2025) 17:277. doi: 10.3390/nu17020277 39861407 PMC11767646

[37] Anilkumar SA Dutta S Aboo S Ismail A. Vitamin D as a modulator of molecular pathways involved in CVDs: Evidence from preclinical studies. Life Sci. (2024) 357:123062. doi: 10.1016/j.lfs.2024.123062 39288869

[38] Raza ML Hassan ST Jamil S Fatima W Fatima M. Nutritional interventions in depression: The role of vitamin D and omega-3 fatty acids in neuropsychiatric health. Clin Nutr. (2025) 45:270–80. doi: 10.1016/j.clnu.2025.01.009 39874718

[39] Pang J Yang C Liu J Wang Z Tao X Cao Z. Correlation between vitamin D metabolic pathway-related gene polymorphisms and cardiovascular disease. Food Funct. (2024) 15:11342–64. doi: 10.1039/d4fo03234a 39494806

[40] Irwin MR. Why sleep is important for health: a psychoneuroimmunology perspective. Annu Rev Psychol. (2015) 66:143–72. doi: 10.1146/annurev-psych-010213-115205 PMC4961463 25061767

[41] Xiang W Lyu K Li Y Yin B Ke L Di Q. Chronic high temperature exposure, brain structure, and mental health: Cross-sectional and prospective studies. Environ Res. (2025) 264:120348. doi: 10.1016/j.envres.2024.120348 39532196

[42] Schiza SE Bouloukaki I. Does gender matter: sex-specific aspects of symptoms, outcome, and therapy of obstructive sleep apnea. Curr Opin Pulm Med. (2020) 26:642–9. doi: 10.1097/MCP.0000000000000728 32890020

[43] Pengo MF Won CH Bourjeily G. Sleep in women across the life span. Chest. (2018) 154:196–206. doi: 10.1016/j.chest.2018.04.005 29679598 PMC6045782

[44] Morssinkhof M van Wylick DW Priester-Vink S van der Werf YD den Heijer M van den Heuvel OA. Associations between sex hormones, sleep problems and depression: A systematic review. Neurosci Biobehav Rev. (2020) 118:669–80. doi: 10.1016/j.neubiorev.2020.08.006 32882313

[45] Im EO Yang YL Liu J Chee W. Sleep-related symptoms of midlife women with and without type 2 diabetes mellitus. Menopause. (2019) 26:1178–84. doi: 10.1097/GME.0000000000001383 PMC7951177 31408021

[46] Chen X Chu C Doebis C von Baehr V Hocher B. Sex-dependent association of vitamin D with insulin resistance in humans. J Clin Endocrinol Metab. (2021) 106:e3739–47. 10.1210/clinem/dgab213 34406392

[47] van den Berg KS Marijnissen RM van den Brink R Oude VR Hegeman JM. Adverse health outcomes in vitamin D supplementation trials for depression: A systematic review. Ageing Res Rev. (2021) 71:101442. doi: 10.1016/j.arr.2021.101442 34390851

[48] Lindberg E Bonsignore MR Polo-Kantola P. Role of menopause and hormone replacement therapy in sleep-disordered breathing. Sleep Med Rev. (2020) 49:101225. doi: 10.1016/j.smrv.2019.101225 31739179

