# Supplementary material for: Association of serum 25-hydroxyvitamin D with sleep disorders in individuals with prediabetes and diabetes: a prospective cohort study
Source: Front Endocrinol (Lausanne). 2025 Apr 1;16:1524368. doi: 10.3389/fendo.2025.1524368 (PMC11996662; doi:10.3389/fendo.2025.1524368)
Supplement: Supplementary file 1 [file DataSheet1.docx]

**Association of Serum 25-Hydroxyvitamin D with Sleep Disorders in Individuals with Prediabetes and Diabetes: A Prospective Cohort Study**

**Supplementary Materials**

**Table of contents**

| **Supplementary Table 1**  Baseline characteristics of the cross-sectional study participants by serum 25(OH)D. |
| --- |
| **Supplementary Table 2**  The relationship between serum 25(OH)D and different types of sleep disorders in cross-sectional study. |
| **Supplementary Table 3**  The relationship between serum 25(OH)D and different types of sleep disorders in prospective study. |
| **Supplementary Table 4**  Hazard ratios (95% confidence intervals) for sleep disorders based on serum 25(OH)D concentrations in individuals with prediabetes and diabetes, excluding individuals who experienced depression within the last two years. |
| **Supplementary Figure 1**  Multivariable adjusted restricted cubic splines of the odds ratios of sleep disorders based on serum 25(OH)D concentrations in individuals with prediabetes and diabetes. |
| **Supplementary Figure 2**  Multivariable adjusted odds ratios (95% confidence intervals) of sleep disorders based on serum 25(OH)D concentrations in individuals with prediabetes, stratified by age, sex, body mass index (BMI), and smoking status. |
| **Supplementary Figure 3**  Multivariable adjusted odds ratios (95% confidence intervals) of sleep disorders based on serum 25(OH)D concentrations in individuals with diabetes, stratified by age, sex, body mass index (BMI), and smoking status. |

**Supplementary Table 1. Baseline characteristics of the cross-sectional study participants by serum 25(OH)D.**

| **Baseline characteristics*** | **Total** | **Serum 25(OH)D levels in prediabetes, nmol/L** | | | | |  | **Serum 25(OH)D levels in diabetes, nmol/L** | | | | |
| --- | --- | --- | --- | --- | --- | --- | --- | --- | --- | --- | --- | --- |
|  |  | **< 25** | **25 to 50** | **50 to 75** | **≥ 75** | **P** |  | **< 25** | **25 to 50** | **50 to 75** | **≥ 75** | **P** |
| No. of participants, n | 81553 | 9794 | 27053 | 19004 | 5807 |  |  | 4485 | 9107 | 4978 | 1325 |  |
| Age, years | 59.6 (7.1) | 57.4 (7.7) | 59.4 (7.0) | 60.6 (6.5) | 61.0 (6.3) | <0.001 |  | 57.7 (7.5) | 59.5 (7.1) | 60.8 (6.6) | 61.0 (6.5) | <0.001 |
| Sex |  |  |  |  |  |  |  |  |  |  |  |  |
| Male | 41275 (50.6%) | 4755 (48.6%) | 12681 (46.9%) | 8816 (46.4%) | 2793 (48.1%) |  |  | 2629 (58.6%) | 5616 (61.7%) | 3174 (63.8%) | 811 (61.2%) |  |
| Female | 40278 (49.4%) | 5039 (51.4%) | 14372 (53.1%) | 10188 (53.6%) | 3014 (51.9%) | 0.002 |  | 1856 (41.4%) | 3491 (38.3%) | 1804 (36.2%) | 514 (38.8%) | <0.001 |
| Body mass index, kg/m2 | 29.6 (5.6) | 30.4 (6.1) | 29.5 (5.4) | 28.3 (4.7) | 27.1 (4.4) | <0.001 |  | 32.6 (6.8) | 31.9 (5.8) | 30.6 (5.3) | 29.0 (4.9) | <0.001 |
| Ethnicity |  |  |  |  |  |  |  |  |  |  |  |  |
| White | 73036 (89.6%) | 7398 (75.5%) | 24418 (90.3%) | 18237 (96.0%) | 5687 (97.9%) | <0.001 |  | 3339 (74.4%) | 7970 (87.5%) | 4696 (94.3%) | 1291 (97.4%) | <0.001 |
| Mixed | 580 (0.7%) | 127 (1.3%) | 195 (0.7%) | 109 (0.6%) | 18 (0.3%) |  |  | 39 (0.9%) | 64 (0.7%) | 23 (0.5%) | 5 (0.4%) |  |
| Asian | 3741 (4.6%) | 1240 (12.7%) | 963 (3.6%) | 210 (1.1%) | 29 (0.5%) |  |  | 726 (16.2%) | 455 (5.0%) | 105 (2.1%) | 13 (1.0%) |  |
| Black | 2578 (3.2%) | 657 (6.7%) | 926 (3.4%) | 255 (1.3%) | 39 (0.7%) |  |  | 211 (4.7%) | 385 (4.2%) | 95 (1.9%) | 10 (0.8%) |  |
| Others | 1618 (2.0%) | 372 (3.8%) | 551 (2.0%) | 193 (1.0%) | 34 (0.6%) |  |  | 170 (3.8%) | 233 (2.6%) | 59 (1.2%) | 6 (0.5%) |  |
| Education** |  |  |  |  |  |  |  |  |  |  |  |  |
| 1 | 20565 (25.2%) | 2720 (27.8%) | 7231 (26.7%) | 4680 (24.6%) | 1350 (23.2%) | <0.001 |  | 1121 (25.0%) | 2150 (23.6%) | 1033 (20.8%) | 280 (21.1%) | <0.001 |
| 2 | 28133 (34.5%) | 3233 (33.0%) | 9303 (34.4%) | 6818 (35.9%) | 2145 (36.9%) |  |  | 1502 (33.5%) | 3028 (33.2%) | 1646 (33.1%) | 458 (34.6%) |  |
| 3 | 11278 (13.8%) | 1292 (13.2%) | 3693 (13.7%) | 2651 (13.9%) | 807 (13.9%) |  |  | 593 (13.2%) | 1317 (14.5%) | 726 (14.6%) | 199 (15.0%) |  |
| 4 | 21577 (26.5%) | 2549 (26.0%) | 6826 (25.2%) | 4855 (25.5%) | 1505 (25.9%) |  |  | 1269 (28.3%) | 2612 (28.7%) | 1573 (31.6%) | 388 (29.3%) |  |
| Household income, ￡ |  |  |  |  |  |  |  |  |  |  |  |  |
| <18,000 | 22023 (27.0%) | 3031 (30.9%) | 7035 (26.0%) | 4463 (23.5%) | 1350 (23.2%) | <0.001 |  | 1599 (35.7%) | 2746 (30.2%) | 1448 (29.1%) | 351 (26.5%) | <0.001 |
| 18000-30,999 | 19314 (23.7%) | 2076 (21.2%) | 6371 (23.6%) | 4759 (25.0%) | 1442 (24.8%) |  |  | 942 (21.0%) | 2104 (23.1%) | 1267 (25.5%) | 353 (26.6%) |  |
| 31,000-51,999 | 14998 (18.4%) | 1719 (17.6%) | 5252 (19.4%) | 3644 (19.2%) | 1113 (19.2%) |  |  | 697 (15.5%) | 1506 (16.5%) | 827 (16.6%) | 240 (18.1%) |  |
| >52,000 | 11113 (13.6%) | 1268 (12.9%) | 3842 (14.2%) | 2787 (14.7%) | 884 (15.2%) |  |  | 450 (10.0%) | 1133 (12.4%) | 585 (11.8%) | 164 (12.4%) |  |
| Others | 14105 (17.3%) | 1700 (17.4%) | 4553 (16.8%) | 3351 (17.6%) | 1018 (17.5%) |  |  | 797 (17.8%) | 1618 (17.8%) | 851 (17.1%) | 217 (16.4%) |  |
| Smoking status |  |  |  |  |  |  |  |  |  |  |  |  |
| Never | 38818 (47.6%) | 4632 (47.3%) | 13165 (48.7%) | 9315 (49.0%) | 2671 (46.0%) | <0.001 |  | 2088 (46.6%) | 4165 (45.7%) | 2197 (44.1%) | 585 (44.2%) | <0.001 |
| Previous | 30631 (37.6%) | 2907 (29.7%) | 9626 (35.6%) | 7312 (38.5%) | 2420 (41.7%) |  |  | 1610 (35.9%) | 3802 (41.7%) | 2327 (46.7%) | 627 (47.3%) |  |
| Current | 11628 (14.3%) | 2182 (22.3%) | 4138 (15.3%) | 2278 (12.0%) | 693 (11.9%) |  |  | 755 (16.8%) | 1067 (11.7%) | 416 (8.4%) | 99 (7.5%) |  |
| Others | 476 (0.6%) | 73 (0.7%) | 124 (0.5%) | 99 (0.5%) | 23 (0.4%) |  |  | 32 (0.7%) | 73 (0.8%) | 38 (0.8%) | 14 (1.1%) |  |
| Drinking status |  |  |  |  |  | <0.001 |  |  |  |  |  | <0.001 |
| Never | 5722 (7.0%) | 1206 (12.3%) | 1691 (6.3%) | 829 (4.4%) | 208 (3.6%) |  |  | 661 (14.7%) | 782 (8.6%) | 298 (6.0%) | 47 (3.5%) |  |
| Previous | 4349 (5.3%) | 580 (5.9%) | 1316 (4.9%) | 752 (4.0%) | 212 (3.7%) |  |  | 411 (9.2%) | 671 (7.4%) | 335 (6.7%) | 72 (5.4%) |  |
| Current | 71296 (87.4%) | 7961 (81.3%) | 23992 (88.7%) | 17397 (91.5%) | 5380 (92.6%) |  |  | 3399 (75.8%) | 7629 (83.8%) | 4334 (87.1%) | 1204 (90.9%) |  |
| Others | 186 (0.2%) | 47 (0.5%) | 54 (0.2%) | 26 (0.1%) | 7 (0.1%) |  |  | 14 (0.3%) | 25 (0.3%) | 11 (0.2%) | 2 (0.2%) |  |
| Sleeping duration,,hours/day |  |  |  |  |  |  |  |  |  |  |  |  |
| ≤6 | 22761 (27.9%) | 3220 (32.9%) | 7685 (28.4%) | 4808 (25.3%) | 1386 (23.9%) | <0.001 |  | 1473 (32.8%) | 2697 (29.6%) | 1206 (24.2%) | 286 (21.6%) | <0.001 |
| 7-8 | 50895 (62.4%) | 5704 (58.2%) | 17091 (63.2%) | 12502 (65.8%) | 3890 (67.0%) |  |  | 2423 (54.0%) | 5280 (58.0%) | 3162 (63.5%) | 843 (63.6%) |  |
| ≥9 | 7897 (9.7%) | 870 (8.9%) | 2277 (8.4%) | 1694 (8.9%) | 531 (9.1%) |  |  | 589 (13.1%) | 1130 (12.4%) | 610 (12.3%) | 196 (14.8%) |  |
| Summer outdoor time,hours/day | 4.1 (2.5) | 3.6 (2.4) | 3.9 (2.4) | 4.3 (2.4) | 4.6 (2.5) | <0.001 |  | 3.7 (2.5) | 4.0 (2.6) | 4.5 (2.5) | 4.5 (2.4) | <0.001 |
| Glucose-lowering drugs | 14412 (17.7%) | 398 (4.1%) | 929 (3.4%) | 555 (2.9%) | 194 (3.3%) | <0.001 |  | 2675 (59.6%) | 5570 (61.2%) | 3147 (63.2%) | 944 (71.2%) | <0.001 |
| Antihypertensive drugs | 18633 (22.8%) | 1839 (18.8%) | 4878 (18.0%) | 3329 (17.5%) | 1060 (18.3%) | 0.064 |  | 1676 (37.4%) | 3503 (38.5%) | 1836 (36.9%) | 512 (38.6%) | 0.237 |
| Lowering-cholesterol drugs | 30302 (37.2%) | 2675 (27.3%) | 7347 (27.2%) | 5365 (28.2%) | 1939 (33.4%) | <0.001 |  | 2723 (60.7%) | 5907 (64.9%) | 3352 (67.3%) | 994 (75.0%) | <0.001 |
| Vitamin assessment seasons |  |  |  |  |  |  |  |  |  |  |  |  |
| Spring | 24436 (30.0%) | 2919 (29.8%) | 8173 (30.2%) | 5666 (29.8%) | 1725 (29.7%) | 0.225 |  | 1324 (29.5%) | 2715 (29.8%) | 1562 (31.4%) | 352 (26.6%) | 0.09 |
| Summer | 24010 (29.4%) | 2804 (28.6%) | 8003 (29.6%) | 5628 (29.6%) | 1714 (29.5%) |  |  | 1344 (30.0%) | 2680 (29.4%) | 1417 (28.5%) | 420 (31.7%) |  |
| Fall | 14635 (17.9%) | 1812 (18.5%) | 4696 (17.4%) | 3447 (18.1%) | 1043 (18.0%) |  |  | 803 (17.9%) | 1694 (18.6%) | 892 (17.9%) | 248 (18.7%) |  |
| Winter | 18472 (22.7%) | 2259 (23.1%) | 6181 (22.8%) | 4263 (22.4%) | 1325 (22.8%) |  |  | 1014 (22.6%) | 2018 (22.2%) | 1107 (22.2%) | 305 (23.0%) |  |
| Vitamin supplement intake | 3281 (4.0%) | 179 (1.8%) | 887 (3.3%) | 1086 (5.7%) | 440 (7.6%) | <0.001 |  | 82 (1.8%) | 281 (3.1%) | 252 (5.1%) | 74 (5.6%) | <0.001 |
| Parathyroid diseases | 86 (0.1%) | 13 (0.1%) | 22 (0.1%) | 14 (0.1%) | 4 (0.1%) | 0.411 |  | 9 (0.2%) | 11 (0.1%) | 9 (0.2%) | 4 (0.3%) | 0.324 |
| Sleep disorders | 1007 (1.2%) | 123 (1.3%) | 243 (0.9%) | 158 (0.8%) | 38 (0.7%) | <0.001 |  | 116 (2.6%) | 210 (2.3%) | 101 (2.0%) | 18 (1.4%) | 0.039 |
| Sleep apnea | 828 (1.0%) | 97 (1.0%) | 201 (0.7%) | 135 (0.7%) | 30 (0.5%) | 0.007 |  | 95 (2.1%) | 170 (1.9%) | 83 (1.7%) | 17 (1.3%) | 0.167 |
| Other sleep disorders | 179 (0.2%) | 26 (0.3%) | 42 (0.2%) | 23 (0.1%) | 8 (0.1%) | 0.032 |  | 21 (0.5%) | 40 (0.4%) | 18 (0.4%) | 1 (0.1%) | 0.209 |

**Supplementary Table 2. The relationship between serum 25(OH)D and different types of sleep disorders in cross-sectional study.**

| **Serum 25(OH)D** | **Sleep apnea** | | | |  | **Other sleep disorder** | | | |
| --- | --- | --- | --- | --- | --- | --- | --- | --- | --- |
|  | **Event/Total (%)** | **OR(95% CI)** | **p overall** | **p for trend** |  | **Event/Total (%)** | **OR(95% CI)** | **p overall** | **p for trend** |
| **Prediabetes(n= 61658)** |  |  |  |  |  |  |  |  |  |
| < 25 | 97/9794(0.99%) | Reference |  | 0.005 |  | 26/9794(0.27%) | Reference |  | 0.024 |
| 25 to 50 | 201/27053(0.74%) | 0.76 (0.59-0.98) | 0.036 |  |  | 42/27053(0.16%) | 0.58 (0.35-0.95) | 0.032 |  |
| 50 to 75 | 135/19004(0.71%) | 0.74 (0.56-0.98) | 0.037 |  |  | 23/19004(0.12%) | 0.45 (0.25-0.82) | 0.008 |  |
| ≥ 75 | 30/5807(0.52%) | 0.53 (0.35-0.81) | 0.004 |  |  | 8/5807(0.14%) | 0.51 (0.22-1.16) | 0.107 |  |
| Per SD | 463/61658(0.75%) | 0.85 (0.77-0.95) | 0.002 |  |  | 99/61658(0.16%) | 0.78 (0.62-0.98) | 0.033 |  |
| **Diabetes(n=19895)** |  |  |  |  |  |  |  |  |  |
| < 25 | 95/4485(2.12%) | Reference |  | 0.059 |  | 21/4485(0.47%) | Reference |  | 0.112 |
| 25 to 50 | 170/9107(1.87%) | 0.87 (0.67-1.14) | 0.313 |  |  | 40/9107(0.44%) | 0.96 (0.55-1.65) | 0.876 |  |
| 50 to 75 | 83/4978(1.67%) | 0.81 (0.59-1.10) | 0.177 |  |  | 18/4978(0.36%) | 0.81 (0.41-1.57) | 0.524 |  |
| ≥ 75 | 17/1325(1.28%) | 0.62 (0.37-1.06) | 0.083 |  |  | 1/1325(0.08%) | 0.17 (0.02-1.29) | 0.087 |  |
| Per SD | 365/19895(1.83%) | 0.86 (0.76-0.96) | 0.01 |  |  | 80/19895(0.40%) | 0.80 (0.62-1.04) | 0.094 |  |

Model : adjusted for age, sex (male, female), ethnicity (White, Mixed, Asian, Black, Others), education (college or university degree, A/AS levels or equivalent or O levels/GCSEs, NVQ or HND or HNC or equivalent or other professional qualifications, Others), house income(£18,000, £18,000-30,999,£31,000-51,999,> £52,000, Others), summer outdoor time (hours/day),vitamin D assessment season (spring, summer, fall, or winter), vitamin D supplements(yes or no), Sleep duration (≤6, 7-8, ≥9 hours/day), Smoking status (Never, Previous, Current, Others), Drinking status (Never, Previous, Current, Others), Glucose-lowering drugs (yes, no). Antihypertensive drugs (yes, no), Lowering-cholesterol drugs (yes, no), Parathyroid diseases (yes, no).

**Supplementary Table 3. The relationship between serum 25(OH)D and different types of sleep disorders in prospective study.**

| **Serum 25(OH)D** | **Sleep apnea** | | | |  | **Other sleep disorder** | | | |
| --- | --- | --- | --- | --- | --- | --- | --- | --- | --- |
|  | **Event/Total (%)** | **OR(95% CI)** | **p overall** | **p for trend** |  | **Event/Total (%)** | **OR(95% CI)** | **p overall** | **p for trend** |
| **Prediabetes(n= 61096)** |  |  |  |  |  |  |  |  |  |
| < 25 | 327/9671(3.38%) | Reference |  | <0.001 |  | 34/9671(0.35%) | Reference |  | 0.034 |
| 25 to 50 | 691/26810(2.58%) | 0.78 (0.68-0.89) | <0.001 |  |  | 92/26810(0.34%) | 0.99 (0.66-1.49) | 0.979 |  |
| 50 to 75 | 408/18846(2.16%) | 0.67 (0.58-0.79) | <0.001 |  |  | 54/18846(0.29%) | 0.84 (0.54-1.32) | 0.46 |  |
| ≥ 75 | 99/5769(1.72%) | 0.53 (0.42-0.67) | <0.001 |  |  | 8/5769(0.14%) | 0.41 (0.19-0.91) | 0.027 |  |
| Per SD | 1525/61096(2.50%) | 0.83 (0.78-0.88) | <0.001 |  |  | 188/61096(0.31%) | 0.85 (0.72-1.00) | 0.045 |  |
| **Diabetes(n=19450)** |  |  |  |  |  |  |  |  |  |
| < 25 | 245/4369(5.61%) | Reference |  | <0.001 |  | 26/4369(0.60%) | Reference |  | 0.004 |
| 25 to 50 | 456/8897(5.13%) | 0.87 (0.74-1.02) | 0.085 |  |  | 33/8897(0.37%) | 0.62 (0.36-1.05) | 0.076 |  |
| 50 to 75 | 182/4877(3.73%) | 0.64 (0.52-0.78) | <0.001 |  |  | 9/4877(0.18%) | 0.32 (0.14-0.69) | 0.004 |  |
| ≥ 75 | 37/1307(2.83%) | 0.49 (0.34-0.69) | <0.001 |  |  | 3/1307(0.23%) | 0.39 (0.12-1.33) | 0.132 |  |
| Per SD | 920/19450(4.73%) | 0.82 (0.76-0.88) | <0.001 |  |  | 71/19450(0.37%) | 0.72 (0.54-0.96) | 0.024 |  |

Model : adjusted for age, sex (male, female), ethnicity (White, Mixed, Asian, Black, Others), education (college or university degree, A/AS levels or equivalent or O levels/GCSEs, NVQ or HND or HNC or equivalent or other professional qualifications, Others), house income(£18,000, £18,000-30,999,£31,000-51,999,> £52,000, Others), summer outdoor time (hours/day),vitamin D assessment season (spring, summer, fall, or winter), vitamin D supplements(yes or no), Sleep duration (≤6, 7-8, ≥9 hours/day), Smoking status (Never, Previous, Current, Others), Drinking status (Never, Previous, Current, Others), Glucose-lowering drugs (yes, no). Antihypertensive drugs (yes, no), Lowering-cholesterol drugs (yes, no), Parathyroid diseases (yes, no).

**Supplementary Table 4. Hazard ratios (95% confidence intervals) for sleep disorders based on serum 25(OH)D concentrations in individuals with prediabetes and diabetes, excluding individuals who experienced depression within the last two years.**

| **Serum 25(OH)D** | **Event/Total (%)** | **Model1** | | |  | **Model2** | | |  | **Model3** | | |
| --- | --- | --- | --- | --- | --- | --- | --- | --- | --- | --- | --- | --- |
|  |  | **HR(95% CI)** | **p overall** | **p for trend** |  | **HR(95% CI)** | **p overall** | **p for trend** |  | **HR(95% CI)** | **p overall** | **p for trend** |
| **Prediabetes(n= 60440)** |  |  |  |  |  |  |  |  |  |  |  |  |
| < 25 | 315/9535(3.30%) | Reference |  | <0.001 |  | Reference |  | <0.001 |  | Reference |  | <0.001 |
| 25 to 50 | 688/26524(2.59%) | 0.78 (0.68-0.89) | <0.001 |  |  | 0.79 (0.69-0.90) | <0.001 |  |  | 0.81 (0.70-0.93) | 0.002 |  |
| 50 to 75 | 415/18668(2.22%) | 0.66 (0.57-0.76) | <0.001 |  |  | 0.68 (0.58-0.79) | <0.001 |  |  | 0.71 (0.61-0.83) | <0.001 |  |
| ≥ 75 | 100/5713(1.75%) | 0.52 (0.42-0.65) | <0.001 |  |  | 0.54 (0.43-0.67) | <0.001 |  |  | 0.56 (0.44-0.70) | <0.001 |  |
| per SD | 1518/60440(2.51%) | 0.83 (0.79-0.87) | <0.001 |  |  | 0.84 (0.79-0.88) | <0.001 |  |  | 0.85 (0.80-0.90) | <0.001 |  |
| **Diabetes(n=19065)** |  |  |  |  |  |  |  |  |  |  |  |  |
| < 25 | 231/4251(5.43%) | Reference |  | <0.001 |  | Reference |  | <0.001 |  | Reference |  | <0.001 |
| 25 to 50 | 424/8720(4.86%) | 0.87 (0.74-1.03) | 0.098 |  |  | 0.84 (0.72-0.99) | 0.042 |  |  | 0.86 (0.73-1.01) | 0.065 |  |
| 50 to 75 | 174/4813(3.62%) | 0.64 (0.53-0.78) | <0.001 |  |  | 0.61 (0.50-0.75) | <0.001 |  |  | 0.64 (0.53-0.79) | <0.001 |  |
| ≥ 75 | 34/1281(2.65%) | 0.47 (0.33-0.68) | <0.001 |  |  | 0.46 (0.32-0.67) | <0.001 |  |  | 0.47 (0.33-0.68) | <0.001 |  |
| per SD | 863/19065(4.53%) | 0.83 (0.77-0.89) | <0.001 |  |  | 0.82 (0.76-0.88) | <0.001 |  |  | 0.83 (0.77-0.90) | <0.001 |  |

Model 1: unadjusted model.

Model 2: adjusted for age, sex (male, female), ethnicity (White, Mixed, Asian, Black, Others), education (college or university degree, A/AS levels or equivalent or O levels/GCSEs, NVQ or HND or HNC or equivalent or other professional qualifications, Others), house income(£18,000, £18,000-30,999,£31,000-51,999,> £52,000, Others), summer outdoor time (hours/day),vitamin D assessment season (spring, summer, fall, or winter), vitamin D supplements(yes or no).

Model 3: adjusted for model 2 variables as well as sleep duration (≤6, 7-8, ≥9 hours/day), Smoking status (Never, Previous, Current, Others), Drinking status (Never, Previous, Current, Others), Glucose-lowering drugs (yes, no). Antihypertensive drugs (yes, no), Lowering-cholesterol drugs (yes, no), Parathyroid diseases (yes, no)

**Supplementary Figure 1. Multivariable adjusted restricted cubic splines of the odds ratios of sleep disorders based on serum 25(OH)D concentrations in individuals with prediabetes and diabetes.**

**
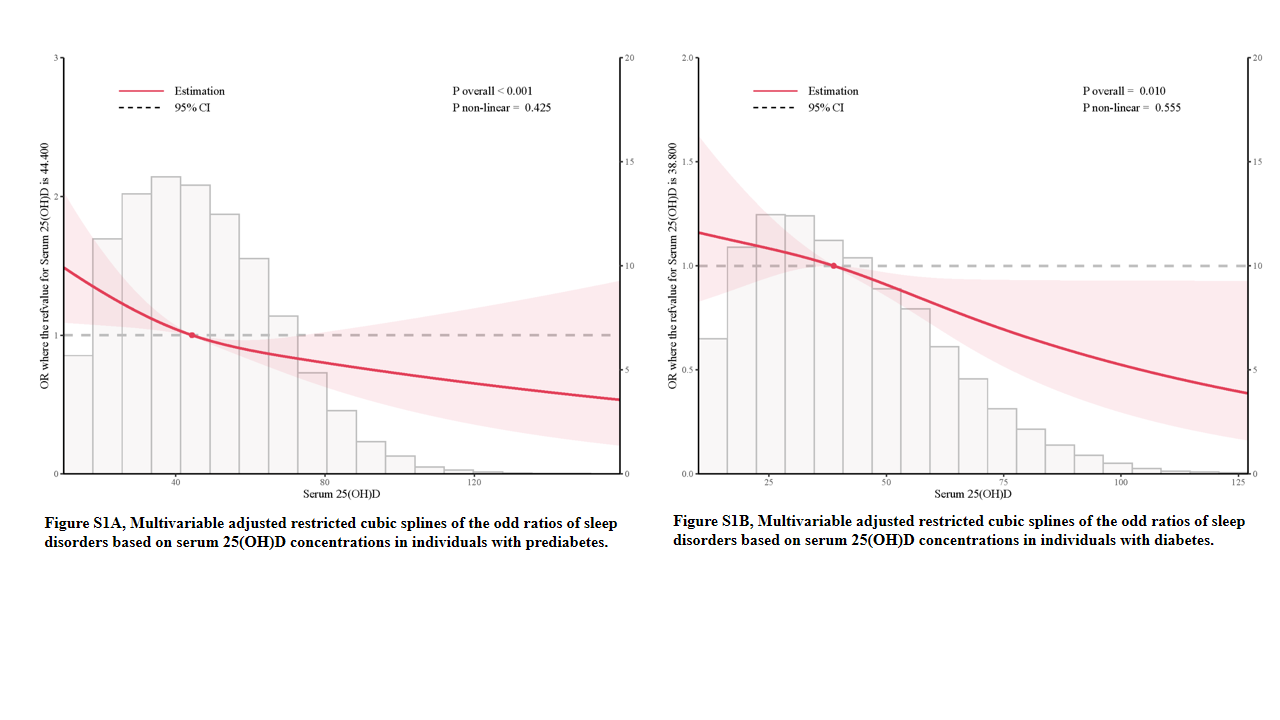
**

**Supplementary Figure 2. Multivariable adjusted odds ratios (95% confidence intervals) of sleep disorders based on serum 25(OH)D concentrations in individuals with prediabetes, stratified by age, sex, body mass index (BMI), and smoking status.**

**
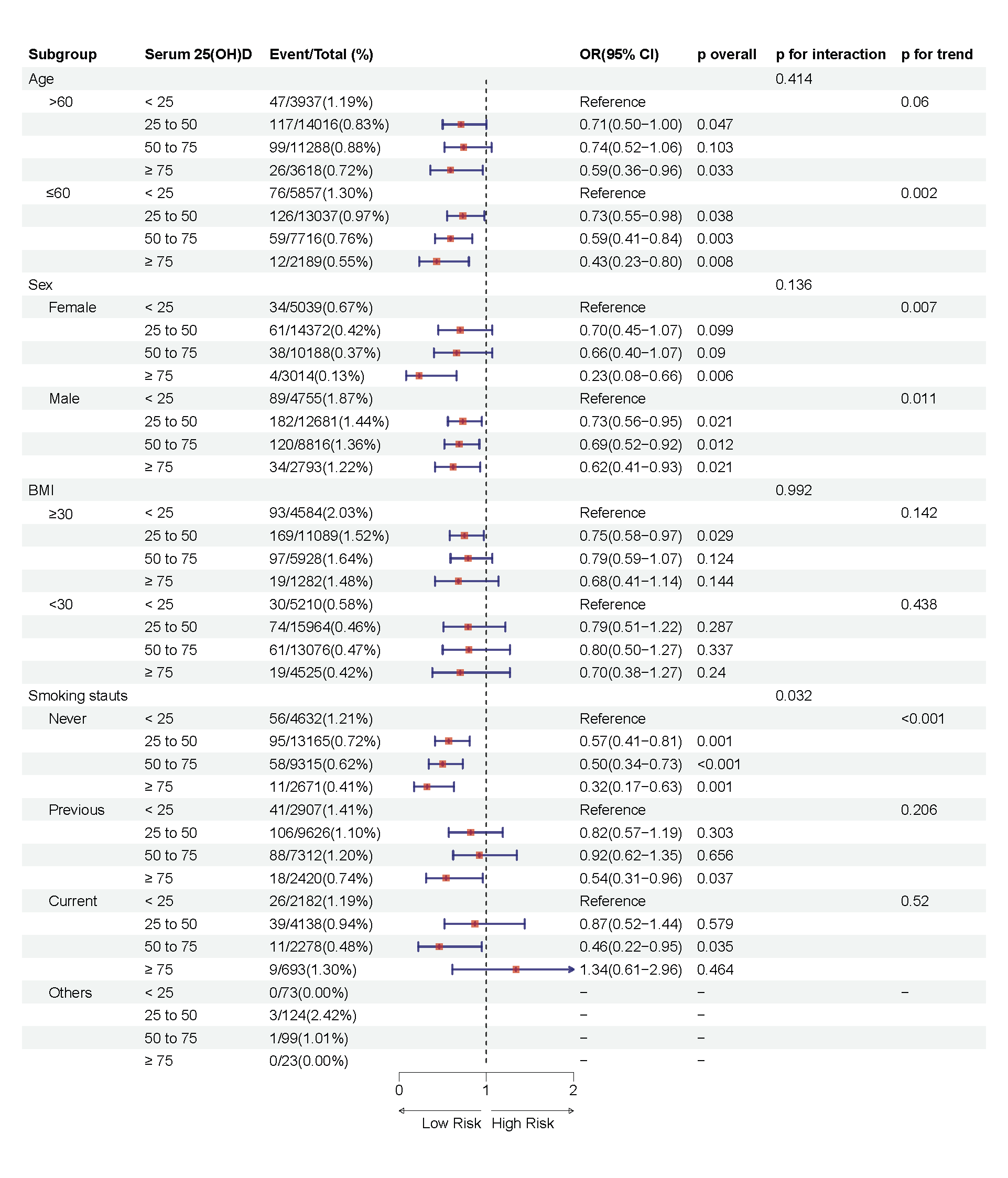
**

**Supplementary Figure 3. Multivariable adjusted odds ratios (95% confidence intervals) of sleep disorders based on serum 25(OH)D concentrations in individuals with diabetes, stratified by age, sex, body mass index (BMI), and smoking status.**

**
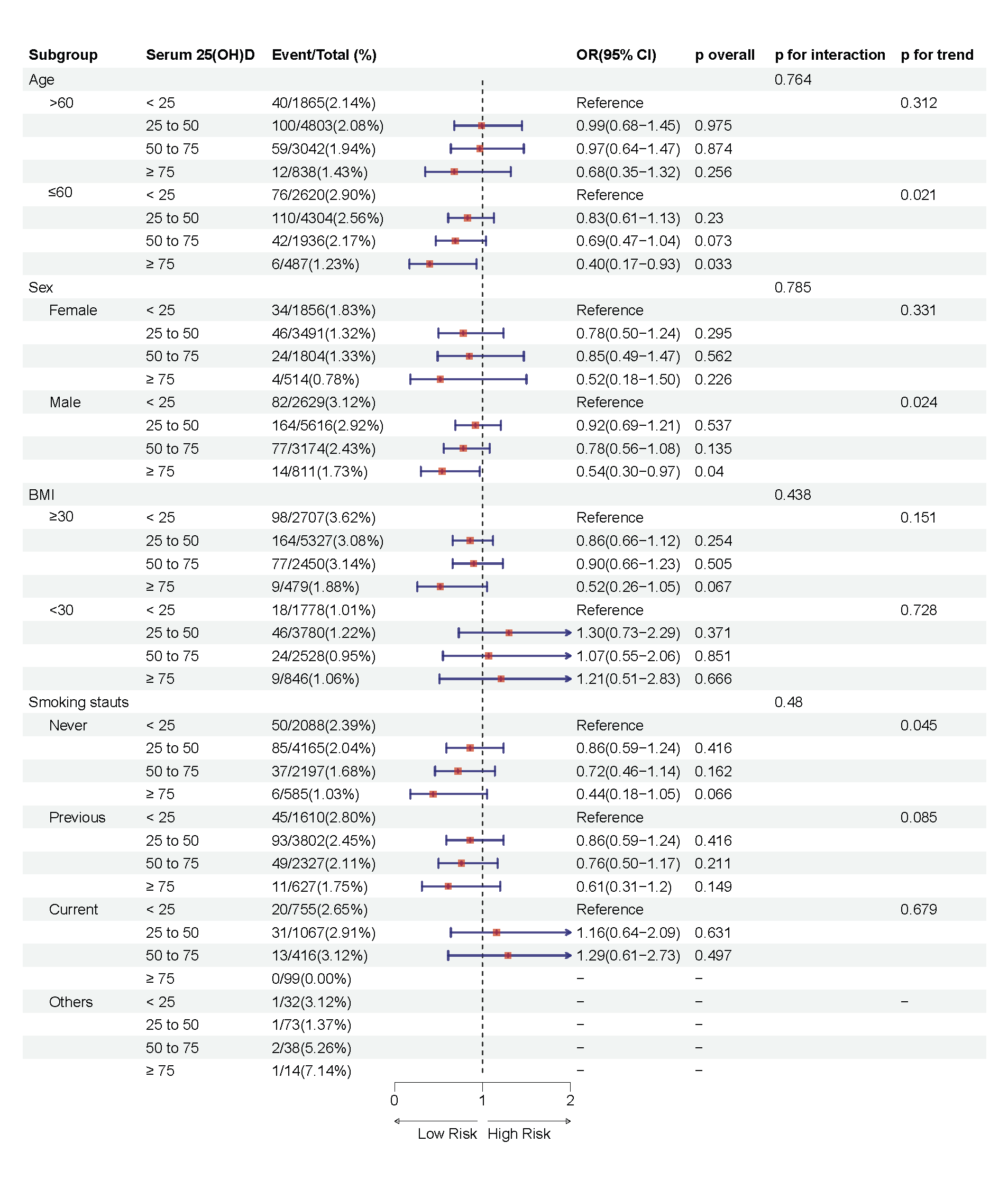
**
